# Stereoregular radical polymers enable selective spin transfer

**DOI:** 10.1126/sciadv.adr4004

**Published:** 2025-03-21

**Authors:** Hyunki Yeo, Cole C. Sorensen, Hamas Tahir, Andrew Marquardt, Yun-Fang Yang, Nick Legaux, Brett M. Savoie, Frank A. Leibfarth, Bryan W. Boudouris

**Affiliations:** ^1^ Charles D. Davidson School of Chemical Engineering, Purdue University, West Lafayette, IN 47907, USA.; ^2^ Department of Chemistry, University of North Carolina, Chapel Hill, Chapel Hill, NC 27599, USA.; ^3^ Department of Chemistry, Purdue University, West Lafayette, IN 47907, USA.

## Abstract

Spintronic devices are emerging as an approach to realize performance and energy efficiency beyond what is possible with traditional electronic devices. State-of-the-art metals and doped conjugated polymers used for spin manipulation suffer from fundamental performance and stability issues. We leveraged stereoselective cationic polymerization to design a polymer with a stable persistent radical in each repeat unit that enables the long-range order necessary for spin transport. This approach overcomes conventional requirements for doping in organic spin-pumping devices while showcasing high conductivity, long spin-diffusion lengths, and processability. Molecular-level alterations in polymer stereochemistry were critical for controlling spin-spin interactions and alignment. Stereoregular polymers with persistent neutral radicals represent a previously unidentified class of materials for manipulating spins over long distances for applications in next-generation information storage.

## Teaser

Stereoregular polymers with persistent radicals transport spins over long distances for next-generation information storage.

## INTRODUCTION

**Fig. 1.**
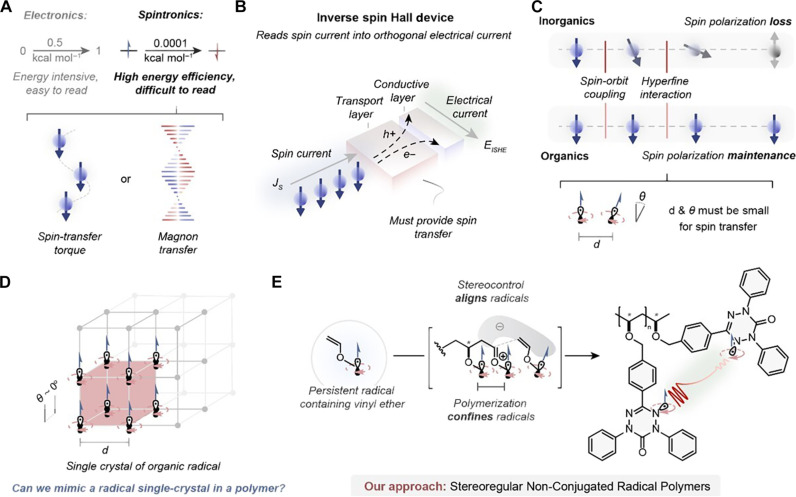
Organic spintronic materials offer improved transport properties. (**A**) Spintronics use the spin of electrons for high-efficiency computing through the manipulation of spin-transfer torque or magnon transfer. (**B**) The ISHE converts a pure spin current to a transverse electrical current for manipulation and reading. (**C**) Transporting spin current is challenging with inorganics but can be better managed by organics if paramagnetic species are in close proximity and aligned. (**D**) Organic radical crystals have the properties of desirable organic spin transport layers but are difficult to process. (**E**) Stereoregular nonconjugated radical polymers combine excellent processability with long-range order of paramagnetic species.

The emerging field of spintronics exploits the quantum spin degree of freedom of electrons to enable computational efficiency beyond the operational limitations imposed by classical physics (Fig. 1A) (*1*–*3*). The operation of spintronic devices rely on the delicate manipulation and transportation of spin currents composed of electron coupled spin-transfer torque or spin waves (i.e., magnons) (Fig. 1A) (*2*, *4*). Reading a spin current can be accomplished by transforming it into a transverse charge current through the inverse spin Hall effect (ISHE) (Fig. 1B) (*5*). Currently, inorganic materials are used in ISHE devices because their strong spin-orbit coupling (SOC) results in efficient conversion of spin current to charge current. However, the strong SOC of these materials also restricts their spin-relaxation and spin-diffusion lengths (e.g., <10 nm), which creates a performance trade-off that can potentially be better managed by organic materials that can achieve spin-relaxation lengths up to the micrometer scale (Fig. 1C). Current best-in-class organic spin conducting materials rely on conjugated polymer backbones with extensive doping to introduce open-shell species or the incorporation of heavy metal atoms into their π-conjugated backbone (*6*). For these diamagnetic π-conjugated polymers, doping is required to circumvent their intrinsically weak spin exchange interactions between open-shell species and successfully transfer the momentum necessary for efficient ISHE, whereas organometallic conjugated polymers still suffer from short spin-relaxation lengths (*7*). In addition, doping and organometallic polymers have spin rectification effects with insufficient voltage outputs due to their heterogeneous-component design that promotes spin backflow at the metal-organic interface (*6*). These intrinsic challenges motivate the development of organic materials that do not require doping, address stability issues, and are paramagnetic to facilitate spin transport.

Nonconjugated radical-containing polymers are emerging as attractive, undoped charge and spin conducting materials due to their enhanced processability, conductivity, and stability (*8*–*10*). These materials transport charge via coupled reduction-oxidation reactions between open-shell species on each repeat unit without the need for chemical doping. The through-space interactions that govern transport in these materials cause them to be highly sensitive to spatial conformation (*11*). While these materials have found substantial success in organic electronics, their implementation in spintronic devices is scarce despite their robust magnetic properties (*9*). The persistent paramagnetic nature of radical polymers should enable their application as spin transport layers, but the lack of long-range order in these amorphous materials has limited their performance in spintronic devices. We identified polymer stereochemistry as a conceptually distinct approach to provide molecular-level control of radical–radical alignment over long distances, with the potential to offer a unique solution to overcome the limitations of spin transport devices made from both inorganic and conjugated organic materials (Fig. 1, C and D) (*12*, *13*).

The direct polymerization of the open-shelled species is necessary for accessing well-defined radical polymers with near quantitative spin incorporation, which is a prerequisite for high-performance spin transfer. Existing nonconjugated radical polymers rely heavily on anionic polymerization, which use harsh reagents and forcing conditions that constrains the scope of radical species amenable to polymerization (*8*, *14*). For example, oxoverdazyl moieties are desirable persistent radicals due to their magnetic properties and ambipolar nature (*15*), but it is not currently possible to polymerize verdazyl-based monomers through anionic polymerization. The cationic polymerization of vinyl ethers represents an attractive approach to access nonconjugated radical polymers for spintronic applications (Fig. 1E). First, the oxidative stability of oxocarbeniums, when sterically protected from Lewis basic functional groups, potentially expands the scope of delocalized open-shell species amenable to polymerization compared to anionic approaches. Second, by taking advantage of the burgeoning field of stereoselective cationic polymerization, we hypothesized that the increased molecular-level ordering in isotactic nonconjugated radical polymers could mitigate spin scattering and improve spin transport.

## RESULTS

**Fig. 2.**
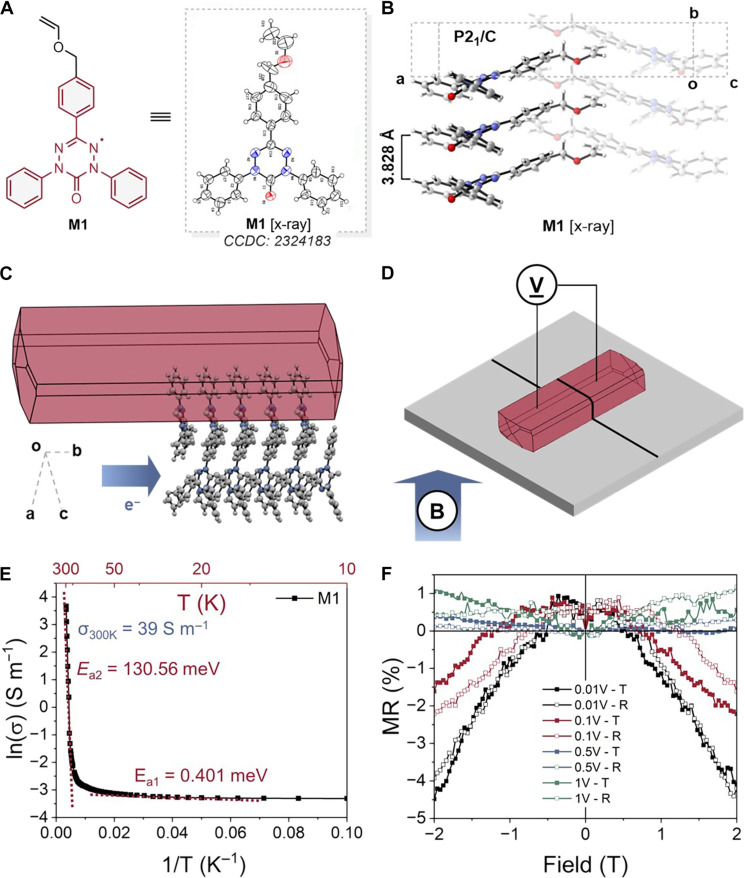
Oxoverdazyl monomer (M1) demonstrates attractive electromagnetic properties. (**A**) Because of its planar chemical structure and structure refinement, **M1** displayed (**B**) a tightly packed pancake bonding structure exhibiting a radical-radical separation of 3.828 Å. (**C**) The calculated Bravais, Friedel, Donnay, and Harker (BFDH) theoretical crystal morphology and a unit cell of **M1** indicates that the charge transfer is occurring through the *b* axis, where the closest packing distance was measured. (**D**) 3D diagram and parameters of single-crystal junction device fabrication with a **M1** single crystal. (**E**) The Arrhenius transport plot obtained from magnetic probe station at zero field. The linear plots indicate the activation energy calculated at two different temperature regions. (**F**) MR results measured from magnetic probe station at 10 K. MR plots shows a trend from negative to positive response as voltage increases (filled square, trace; empty square, retrace).

Our initial studies targeted the synthesis of oxoverdazyl-containing benzyl vinyl ether **M1** given the exceptional stability and delocalized spin character of oxoverdazyls (*16*, *17*). **M1** was synthesized on a gram scale from simple precursors in four steps, and its structure was confirmed by x-ray diffraction (Fig. 2A). The crystal structure of **M1** exhibited pancake bonding (*18*) and a small radical–radical spacing (3.828 Å) in the solid state (Fig. 2B), which suggested its potential for intimate packing and through-space interactions between radicals (Fig. 2C) (*19*–*21*). A single-crystal junction device of **M1** was fabricated and displayed a room temperature conductivity of 39 S m^−1^, which is the highest conductivity measured in a nonconjugated radical conductor to date (Fig. 2, D and E). Charge transport in **M1** follows the Esfros-Shklovskii’s variable range hopping model, indicating the density of states near the Fermi level diminish following a power law (fig. S13), coinciding with an exponential increase in conductivity with temperature (Fig. 2E). Furthermore, the magnetic properties and ability for spin ordering were evaluated for **M1** using magnetoresistance (MR) device measurements. **M1** presented a −4.5% MR value when a 2-T field was applied at 10 K (Fig. 2F), and substantial coupling between the external magnetic field and the orientation of spins was observed. The magnetoresponsivity switched from positive to negative as higher absolute voltages were applied and generally the positive region MR responses were smaller (*22*). This observation is consistent with “spin blockade effect,” by suppressed spin mixing, therefore, leading to reduced current and increased MR (*23*–*26*).

**Fig. 3.**
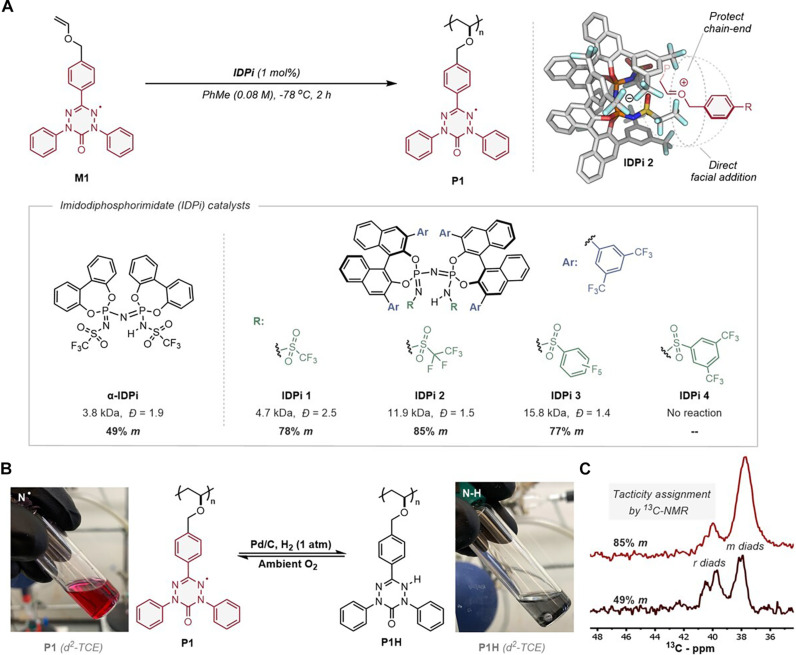
Controlling polymer stereochemistry using sterically bulky IDPi catalysts. (**A**) The highly confined nature of IDPi catalysts enabled the stereoselective polymerization of oxoverdazyl containing monomer (**M1**) to its corresponding polymer (**P1**). A depiction of the posited ion-pairing interaction between **IDPi 2** and growing **P1**. (**B**) The Pd/C catalyzed reduction of poly(oxoverdazyl) **P1** with H_2_ to yield the nonparamagnetic poly(leuco-verdazyl)s **P1H**. (**C**) ^13^C-NMR analysis of isotactic (85% *m*) and atactic (49% *m*) poly(leuco-verdazyl)s’ diad resonances.

Given the importance of intermolecular spin-spin alignment on spin transfer, we pursued the stereoselective polymerization of **M1** through asymmetric ion-pairing catalysis (*27*) to orient the sequential pendant open-shell species along the polymer backbone. Imidodiphosphorimidate (IDPi) Brønsted acids proved to be privileged for this polymerization, presumably because the highly confined counteranion shielded the reactive oxocarbenium chain end from deleterious reactivity and directed enantiofacial monomer addition to enable stereocontrol (Fig. 3A) (*28*–*32*). A series of IDPi catalysts were prepared, which included the achiral 4,4′-biphenol Brønsted acid (**α-IDPi**) and the chiral 1,1′-bi-2-naphthol-based IDPis (**IDPi 1–4**). **M1** was subjected to polymerization at −78°C in PhMe with **α-IDPi** to yield **P1** at a modest molar mass (Fig. 3A). Traditional Lewis and Brønsted acidic cationic polymerization catalysts (*33*–*36*) were unable to polymerize **M1**, demonstrating the unique attributes of the **IDPi** catalyst (fig. S14). Characterization of paramagnetic **P1** required reduction of the oxoverdazyl radical to its leuco-verdazyl form **P1H**, which was accompanied by a loss of its characteristic vibrant red color (Fig. 3B). Integration of the ^13^C–nuclear magnetic resonance (NMR) backbone methylene resonances revealed **P1** to be an atactic polymer, as expected (Fig. 3C). In contrast, **IDPi 1** provided a similar molecular weight and a substantial improvement in stereoregularity to 78% *meso* diads (% *m*). **IDPi 2**, which has a pentafluoroethyl substitution on the sulfonamide, leads to an increase in stereoregularity (85% *m*) and molar mass of the product polymers while also narrowing the dispersity, indicating less chain transfer events. The pentafluorophenylsulfonyl substituted **IDPi 3** resulted in modest tacticity (77% *m*) but with improved molar mass, whereas 3,5-bis(trifluoromethyl)phenylsulfonyl substituted **IDPi 4** displayed no activity. This synthetic campaign represents only the second cationic (*14*) and first stereoselective polymerization of an open-shelled monomer.

**Fig. 4.**
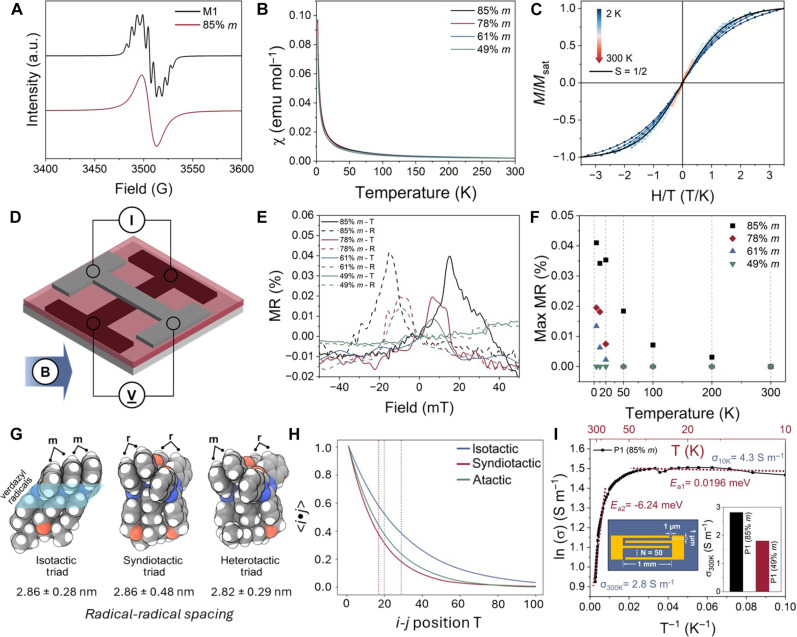
Experimental and computational studies of the electromagnetic character and magnetoelectronic response of stereoregular oxoverdazyl polymers. (**A**) EPR signal comparison of **M1** (black, top) and **P1–**85% *m* (red, bottom). (**B**) χ versus T spectra obtained from SQUID spectroscopy. All the synthesized polymers show paramagnetic behavior. (**C**) Magnetism plots of **P1–**85% *m*. All four polymers follow the Brillouin function models of *S* = 1/2. (**D**) 3D diagram and parameters of spin valve device fabrication. (**E**) MR results of spin-valve devices at 4 K depending on tacticity (filled square, trace sweep; empty square, retrace sweep). (**F**) Maximum MR values measured at various temperatures. (**G**) Lowest energy triad conformers of the ensemble. The isotactic sequence displays increased radical-radical alignment. (**H**) Probabilistic extension of triad sequences exhibiting an increase in radical alignment persistence length in the isotactic polymer. (**I**) Temperature dependence on polymer conductivity with a schematic illustration of an interdigitated 1-μm channel device.

Electron paramagnetic resonance (EPR) spectroscopy confirmed that the radical groups were not quenched during the polymerizations (Fig. 4A). The EPR signal of the molecular radical, **M1**, displays nine resonances due to hyperfine splitting, as expected for an oxoverdazyl, with a nearly 100% integrated intensity compared to a 4-hydroxy-2,2,6,6-tetramethylpiperidine-1-oxyl standard (fig. S15, A and B). Once **M1** is polymerized to create **P1**, the EPR signal transforms to a Lorentzian curve, due to intrachain spin-spin interactions (Fig. 4A). Organic molecules typically display little deviation from the *g* value of a free electron (2.0023) because of their weak SOC. Likewise, the *g* factors between **M1** and **P1** remained unchanged at ~2.003. The radical content in all polymers remained greater than 95%, confirming the selectivity of **IDPi** catalysts for polymerization over radical-terminating side reactions (fig. S15, C and D). **M1** and **P1**s ranging from 49 to 85% *m* display paramagnetic behavior. **P1** follows the Curie-Weiss law throughout the measured temperature range (Fig. 4B and eq. S1) (*37*), while **M1** presents similar paramagnetic behavior (fig. S16). Bulk magnetization measurements of **P1** were modeled using a Brillouin function (eq. S3) and fit for an *S* value of 0.5. This behavior is consistent with a single unpaired electron per oxoverdazyl unit (Fig. 4C and fig. S17) (*38*).

To evaluate the hypothesis that polymer stereochemistry influences the spatial orientation of repeat units in the solid state, and thus spin-spin interactions, spin valves that use **P1** as interfacial layers within a vertical sandwich structure were fabricated to quantify the giant MR (GMR) of the polymers (*39*, *40*). This structure comprised a thin film of **P1** spin-coated between a lanthanum strontium manganite layer and a cobalt (Co) surface (Fig. 4D). The device was cooled to 4 K in the absence of a magnetic field, and resistance measurements were taken as the magnetic field was swept between −50 mT ≤ *B* ≤ 50 mT (table S2 and fig. S18). When the coercive fields of LMSO and Co were oriented antiparallel, no GMR was measured in the atactic **P1**–49% *m*. However, when the isotacticity of the materials was increased, a GMR was observed that scaled with stereoregularity and reached a maximum GMR of 0.04% for the most isotactic **P1–**85% *m* (Fig. 4E). As temperature increased, all of **P1** showed a trend of gradually decreasing MR (Fig. 4F) (*41*). Powder x-ray diffraction provided further information about how tacticity influences the intrachain orientation of oxoverdazyl repeat units (fig. S21). The atactic and moderately isotactic variants of **P1** were amorphous, whereas **P1**–85% *m* demonstrated subtle reflections indicative of a semicrystalline polymer. This onset of crystallinity did not coincide with improved performance and suggests that the stereo-induced GMR is due to molecular orientation and intramolecular effects.

## DISCUSSION

The impact of tacticity on the alignment of the oxoverdazyl repeat units was studied by performing conformational metadynamics sampling of the isotactic, syndiotactic, and heterotactic triads (Fig. 4G). The radical alignment was calculated as the normalized dot product of the vector connecting a consistently chosen pair of adjacent bonded nitrogen atoms in each radical. Each of these quantities was Boltzmann weighted on the basis the energy of its conformation according to

$$<x>=\sum_{i}x_{i}p_{i}=\sum_{i}\frac{x_{i}e^{-\beta E_{i}}}{\sum_{j}e^{-\beta E_{j}}}$$ (1)

where *p_i_* is the relative probability of the observation, *x_i_* is the observed value, *E* is the conformer energy, β is the Boltzmann factor calculated at room temperature, and the summations run over all conformations.

The results of these simulations revealed a strong dipolar alignment facilitated in the *mm* triad, with only the isotactic triad able to consistently align both pairs of radicals. Conversely, the syndiotactic and heterotactic triads align one pair of radicals at the expense of the second. This is reflected in the isotactic triad quantitatively exhibiting the highest probability for alignment (fig. S22). The minimum energy conformation of the *mm* triad (Fig. 4G) also shows a notable similarity to the **M1** pancake bonding crystal structure (Fig. 2B). To calculate the overall effect on the radical alignment persistence length, we performed a probabilistic extension of the triads based on their conformational statistics while enforcing *mr*, *rr*, and *mm* correlations in the alignment statistics (Fig. 4H). The radical alignment decay curves reveal a 53% increase in the radical persistence length in the isotactic polymer (29 repeat units) versus the atactic (19 repeat units) polymer, implying stereo-induced long-range spin-spin alignment between neighboring radicals, which may promote the GMR observed experimentally for **P1**.

Calculating the average radical-radical separation revealed a strong propensity for the radicals to associate, regardless of tacticity, to distances smaller than observed in the single crystal of **M1** (Fig. 4G). The limited effect of tacticity on radical-radical separation was corroborated by a small change in the in-plane conductivity of **P1** between an atactic (1.5 S m^−1^) and isotactic (2.8 S m^−1^) sample (Fig. 4I). Unlike previously reported nonconjugated radical polymers (*8*), annealing above the glass transition was unnecessary for reaching high conductivity, potentially due to the inherently strong radical association of electronically stabilized oxoverdazyls. Notably, a change in charge transport mechanism was observed as the conductivity-temperature relationship of **P1** behaved as a semiconductor below 25 K, but a conductor above (Fig. 4I and fig. S24), which diverges from **M1** and other known organic materials.

**Fig. 5.**
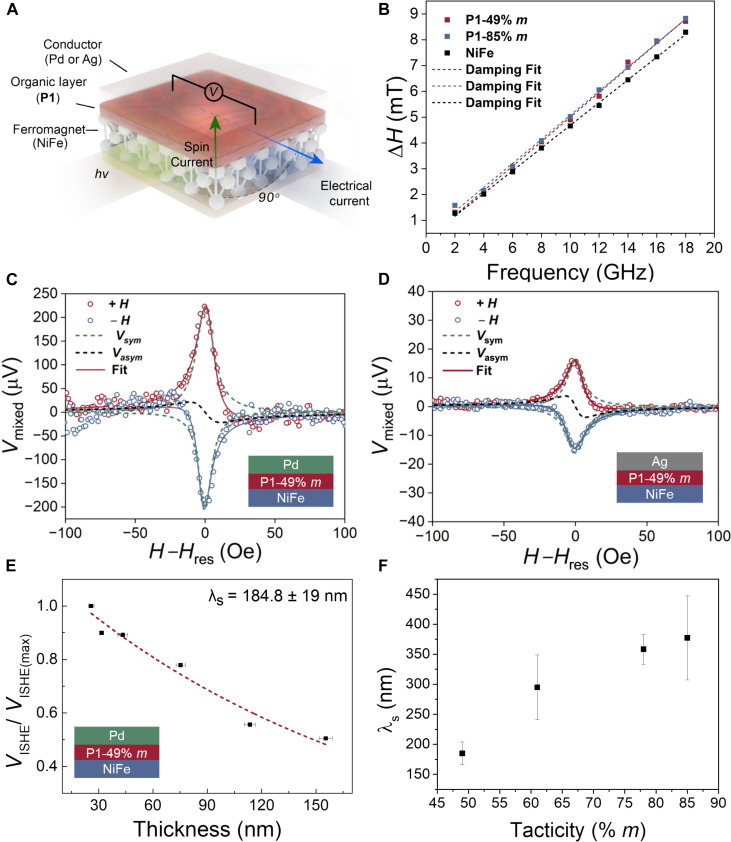
Spin pumping devices for understanding spin transport. (**A**) Device schematics for studying pure spin transport through **P1** with a trilayer structure. (**B**) FMR Linewidth versus frequency response for **P1–**49% *m*, **P1–**85% *m*, and pure NiFe electrode fitted to Eq. 2. (**C**) ISHE response for **P1**–49% *m* at 4 GHz with Pd as spin sink layer. (**D**) ISHE response for **P1**–49% *m* at 4 GHz with Ag as a spin sink layer (**E**) *V*_ISHE_ versus thickness for **P1**–49% *m* fitted to exponential decay function to extract spin diffusion length. (**F**) Plot of tacticity versus spin diffusion length (λs) measured at 4 GHz extracted from exponential decay functions with error bars indicating 2-time SD (*P* value <0.05).

Although spin-valve devices offer valuable insights into spin-dependent transport phenomena, spin-pumping devices are free from conductivity mismatch challenges because they inject magnons, spin polarization waves, rather than spin-polarized charge carriers. Spin-valve devices thus provide a robust testbed for establishing the importance of paramagnetic centers in sustaining pure spin currents. Long spin diffusion lengths (λ_s_) and Gilbert damping parameter (α_eff_) were observed for both **P1–**49% *m* and **P1–**85% *m* at room temperature, showcasing successful magnon injection at FM/**P1** interface. Polymer stereochemistry had little effect on the magnitude of response; however, α_eff_ inverted from positive to negative voltages when comparing 49% to 85% *m*, suggesting that tacticity plays a role in orientating spin-spin interactions at the FM/**P1** interface (see the Supplementary Materials). These measurements were accomplished with a trilayer geometry of NiFe/**P1**/Pd that were mounted on a coplanar waveguide that generates the necessary excitation field for ferromagnetic resonance spectroscopy (FMR) (fig. S25). Microwave excitation in NiFe generates a pure spin current that transports through the **P1** layer and is subsequently converted to charge current in Pd spin sink layer (Fig. 5A). Significant broadening in the peak-to-peak linewidth was observed for both samples, indicating successful transfer of spin angular momentum at the NiFe/**P1** interface. To quantify the momentum leakage, α_eff_ was estimated from the slope of the FMR linewidth (Δ*H*) versus frequency data (Fig. 5B) by fitting to Eq. 2

$$\Delta H=\Delta H_{0}+\frac{\alpha_{\text{eff}}4\pi f}{\;\gamma }$$ (2)

Here, Δ*H*_0_ denotes the inhomogeneous broadening due to structural imperfections, *f* is the microwave frequency, and γ is the gyromagnetic factor. From these measurements, α_eff_ values of 7.0 × 10^−3^, 7.1 × 10^−3^ and 6.6 × 10^−3^ for **P1**–49% *m*, **P1–**85% *m*, and pristine NiFe, respectively, were obtained. The enhancement in damping parameters for both **P1** polymers as compared to pure NiFe thin film indicates that additional leakage or dissipation happens at the interface, which leads to the flow of pure spin current (*J*_s0_) into **P1** by radical-radical exchange interactions and is then completely relaxed at the spin sink layer as detected by an electrical current. The magnetic anisotropy of FM thin film is not significantly altered by **P1** as observed by the frequency versus field response (fig. S29A), and other parameters such as effective spin mixing conductance (*g*_eff_^⇅^) at the NiFe/**P1** interface can be readily calculated (see the Supplementary Materials).

FMR spectroscopy measurements serve as the prerequisite for the ISHE as they inform the resonance field, damping parameter, and saturated magnetization. The maximum voltage is observed at the resonant field and is well defined by the following Lorentzian function

$$V_{\text{mixed}}=V_{\text{sym}}\left[\frac{{\left(\Delta H\right)}^{2}}{4{\left(H-H_{\text{res}}\right)}^{2}+{\left(\Delta H\right)}^{2}}\right]+V_{\text{asym}}\left[\frac{2\Delta H\left(H-H_{\text{res}}\right)}{4{\left(H-H_{\text{res}}\right)}^{2}+{\left(\Delta H\right)}^{2}}\right]$$ (3)

Using the linewidth values,ΔH, determined from FMR, the data in Fig. 5C were fit to Eq. 3 to account for the voltage arising due to the spin-pumping effect (*5*). The first term *V*_sym_ in Eq. 3 accounts for spin pumping effect, while second term *V*_asym_ describes voltage arising from artifact and anisotropic magnetoresistance. While the pure spin current signal is highly symmetric in nature, spin rectification, spin Seebeck, and other Nernst and anomalous Hall effects from NiFe could mask the spin pumping signal. In Fig. 5C, the black dashed curve shows the *V*_sym_ contribution, and the red dashed curve shows the *V*_asym_ contribution. The data fit well to the *V*_sym_ rather than *V*_asym_ component for **P1–**49% *m* with *V*_sym_*/V*_asym_ ratio of ~8, indicating the dominating contribution of spin pumping signal in the overall voltage observed. To further clarify the spin pumping effect in **P1**, the direction of applied negative field was changed from positive to negative. The change in field direction inverts the voltage sign, which is a clear indication of spin pumping effect. To exclude contributions from Pd, the large spin Hall angle spin sink contact Pd was replaced with low spin Hall angle Ag, and a voltage signal with reduced amplitude is still observed (Fig. 5D). This highlights the importance of P1 in transporting pure spin currents without significant relaxation and further supports it as an undoped inverse spin Hall active material. By fitting data into an exponential decay function (eq. S29), a long spin diffusion length (λ_s_) of 184.8 nm was determined in **P1**–49% *m* (Fig. 5E). Furthermore, an increasing trend in λ_s_ was observed with higher isotacticity, reaching a maximum of 377.4 nm for **P1**–85% *m*, approximately twice the value of **P1**–49% *m* (Fig. 5F). The spin diffusion lengths of **P1**–85% *m* are substantially larger than reported doped conjugated polymers such as poly(3,4-ethylenedioxythiophene) (40 nm) (*4*) and within an order of magnitude of graphene (~1 μm) (*42*) with similar device geometries, demonstrating the long-distance spin transport ability of nonconjugated radical organic materials. Computational simulations support the increase λ_s_ as only the isotactic triad can consistently align both pairs of radicals over longer persistence lengths (Fig. 4G). Measurements on the monomer **M1** were also conducted, and although ISHE signals were detected, no thickness-dependent trend was observed, indicating that **M1** functions merely as a charge transport layer without spin polarization (fig. S30). This difference in λ_s_ highlights the key role of radical-radical alignment in transporting pure spin-currents and achieving long-distance spin transport desirable for practical spintronic operation (fig. S31).

Overall, these results showcase that controlling polymer stereochemistry improves radical-radical alignment in nonconjugated polymers, which leads long-distance transfer of spin angular momentum transfer without the need for doping. We anticipate that the identification of molecular-level design concepts to manipulate electron spins over long distances will advance the discovery of spintronic materials, with implications in next-generation information storage and computing.

## MATERIALS AND METHODS

### General considerations

Unless otherwise noted, solvents were dried and degassed using a Pure Process Technology solvent purification system and then subsequently stored over molecular sieves (3 Å) in a N_2_-filled glovebox. The chemical 4-(hydroxymethyl)benzaldehyde was purchased from Ambeed and used received. The chemical *p*-benzoquinone was purchased from Sigma-Aldrich and sublimated before use. Other reagents whose syntheses are not described in Results were purchased from commercial sources [Alfa Aesar (Ward Hill, MA), MilliporeSigma (St. Louis, MO), Oakwood Products (West Columbia, SC), Acros Organics (Geel, Belgium), and TCI America (Portland, OR)] and used without further purification. All syntheses were performed under inert atmosphere (N_2_ or Ar) using flame-dried or oven-dried glassware unless specified otherwise. Qualitative thin-layer chromatography (TLC) analysis was performed on 250-mm-thick, 60-Å, glass-backed F254 silica (SiliCycle, Quebec City, Canada). Visualization was accomplished with ultraviolet light, exposure to p-anisaldehyde solution followed by heating, or exposure to KMnO_4_ solution followed by heating. Flash chromatography was performed using SiliCycle silica gel (230 to 400 mesh). NMR spectra were recorded using a Bruker DRX 400 MHz, Bruker AVANCE III 500 MHz, or Bruker AVANCE III 600 MHz CryoProbe spectrometer. Chemical shifts δ [parts per million (ppm)] are referenced to tetramethylsilane using the residual solvent as an internal standard (^1^H and ^13^C). For ^1^H NMR: CDCl_3_, 7.26 ppm and C_2_D_2_Cl_4_, 6.03 ppm. For ^13^C NMR: CDCl_3_, 77.16 ppm and C_2_D_2_Cl_4_, 73.78 ppm. Coupling constants (J) are expressed in hertz (Hz). High-resolution mass spectrometry was performed with a ThermoScientific Q Exactive HF-X mass spectrometer using electrospray ionization at the University of North Carolina Mass Spectrometry Core Laboratory. Infrared (IR) spectra were obtained using a PerkinElmer Frontier Fourier transform IR (FT-IR) spectrometer. EPR spectra were acquired at room temperature using a Bruker EMXEPR spectrometer. To make the solutions for EPR spectroscopy measurements, monomers and polyoxidovanadates were dissolved in anhydrous chloroform at a concentration of 1 mg ml^−1^. X-ray diffraction spectroscopy was demonstrated by a panalytical Empyrean powder x-ray diffractometer. A high-speed PIXcel 3D Medipix detector was used for analysis of powders.

### Macromolecular characterization

Size exclusion chromatography for samples were performed on an Agilent 1260 Infinity separation module liquid chromatograph equipped with two Agilent Resipore Columns (PL1113- 6300) maintained at 30°C, and an Agilent 1260 RID G1362A refractive index detector at 50°C. A solution of chloroform was used as the mobile phase at a flow rate of 1.0 ml/min. Molecular weight and dispersity data are reported relative to poly(styrene) standards. Decomposition onset temperatures (*T*_d_) of precipitated and dried polymer samples were measured by thermal gravimetric analysis on a TA Instruments Q5000 Thermogravimetric Analyzer. Polymer samples were heated from ambient temperatures to 600°C at a heating rate of 10°C/min. Values of *T*_d_ (temperature at 5% weight loss) were obtained from (wt %) versus temperature (°C) plots. Melting-transition temperature (*T*_m_) and glass-transition temperature (*T*_g_) of precipitated and dried polymer samples were measured using differential scanning calorimetry (DSC) on a TA Instruments Discovery DSC at 10°C/min.

### Synthesis and characterization data (see the Supplementary Materials for structures and schemes)

The IDPi catalysts used herein were prepared according to literature and stored in a glovebox freezer, and were prerepared according to literature (*27*, *30*, *43*). BINOL/biphenol (2 equiv, 698 μmol), sulfonyl phosphorimidoyl trichloride reagent (2 equiv, 698 μmol), and PhMe (7 ml) were added to a 25-ml Schlenk tube (a vessel to allow a slight build-up of pressure) equipped with a magnetic stir bar in a glove box under inert atmosphere (N_2_). Triethylamine (565 mg, 778 μl, 16 equiv, 5.59 mmol) was added all at once and the resultant opaque yellow solution was allowed to stir for 30 min at ambient temperature in the glove box. *N*,*N*-dimethylpyridin-4-amine (21.3 mg, 0.5 equiv, 175 μmol) followed by ammonia (5.94 mg, 970 μl, 0.36 molar, 1 equiv, 349 μmol) in dioxane solution was added to the reaction mixture in quick succession, the reaction vessel was sealed, and the mixture was allowed to stir for 10 min at ambient temperature in the glove box. The closed Schlenk tube containing the reaction mixture was removed from the glove box, heated to 140°C in a silicon oil bath, and stirred for 4 days. After 4 days, the now cloudy yellow reaction was allowed to cool to room temperature and was diluted with EtOAc (10 ml). The diluted reaction mixture was filtered through celite and purified via flash chromatography (9:1 hexanes/EtOAc to 1:1 hexanes/EtOAc). After purification, the catalyst was acidified by stirring in a biphasic solution of 6 M HCl(aq)/CH_2_Cl_2_ (20 and 20 ml respectively) for 2 hours. The layers were separated and concentrated in vacuo. To prevent the IDPi from being inactivated, rather than using a drying agent, the residual water was removed by stripping with dry toluene (5 ml, 3×). The product was obtained as a white solid (see the Supplementary Materials for chemical structure).

### 1,1,1-Trifluoro-*N*-(6-((6-((trifluoromethyl)sulfonamido)-6l5-dibenzo[d,f][1,3,2]dioxaphosphepin-6-ylidene)amino)-6l5-dibenzo[d,f][1,3,2]dioxaphosphepin-6-ylidene)methanesu-lfonamide (a-IDPi)

^1^H NMR (400 MHz, CDCl_3_) δ (ppm): 7.53 (d, *J* = 7.5 Hz, 4H), 7.32–7.41 (m, 8H), and 7.11 (d, *J* = 7.9 Hz, 4H); ^13^C NMR (126 MHz, CDCl_3_) δ (ppm): 146.8 (t, *J* = 4.7 Hz), 130.4, 130.2, 127.7, 127.4, 121.4, and 119.8 (q, *J* = 317.9 Hz); ^19^F NMR (376 MHz, CDCl_3_) δ (ppm): −77.9; ^31^P NMR (162 MHz, CDCl_3_) δ (ppm): −4.97.

### *N*-((11bS)-4-(((11bS)-2,6-bis(3,5-bis(trifluoromethyl)phenyl)-4-(((trifluoromethyl)sulfonyl)imino)-4l5-dinaphtho[2,1-d:1′,2′-f][1,3,2]dioxaphosphepin-4-yl)imino)-2,6-bis(3,5-bis(trifluoromethyl)phenyl)-4l5-dinaphtho[2,1-d:1′,2′-f][1,3,2]dioxaphosphepin-4-yl)-1,1,1-trifluoromethanesulfonamide (IDPi 1)

^1^H NMR (400 MHz, CDCl_3_) δ 8.11 (s, 2H), 8.10–8.06 (m, 2H), 7.97–7.88 (m, 4H), 7.85 (s, 4H), 7.81–7.70 (m, 4H), 7.69–7.55 (m, 6H), 7.43–7.34 (m, 2H), 7.33 (s, 4H), 7.16 (d, *J* = 8.8 Hz, 2H), and 6.65 (s, 2H); ^13^C NMR (100 MHz,CDCl_3_) δ 143.8, 143.73, 143.68, 141.80, 141.75, 141.7, 138.0, 137.8, 133.2, 132.2, 132.0 (q, JC-F = 33.3 Hz), 131.99, 131.84 (q, JC-F = 36.0 Hz), 131.80, 131.2, 130.8, 30.5, 130.0, 129.5, 129.3, 128.8, 128.1, 127.3, 126.8, 123.7, 123.2 (q, JC-F = 271.0 Hz), 123.0 (q, JC-F = 271.5 Hz), 122.0, 121.4, and 118.8 (q, JC-F = 318.7 Hz); ^19^F NMR (376 MHz, CDCl_3_) δ −62.4 (s, 12F), −63.1 (s, 12F), and −79.2 (s, 6F); ^31^P NMR (162 MHz, CDCl_3_) δ −14.9.

### *N*-((11bS)-4-(((11bS)-2,6-bis(3,5-bis(trifluoromethyl)phenyl)-4-(((perfluoroethyl)sulfonyl)imino)-4l5-dinaphtho[2,1-d:1′,2′-f][1,3,2]dioxaphosphepin-4-yl)imino)-2,6-bis(3,5-bis(trifluoromethyl)phenyl)-4l5-dinaphtho[2,1-d:1′,2′-f][1,3,2]dioxaphosphepin-4-yl)-1,1,2,2,2-pentafluoroethane-1-sulfonamide (IDPi 2)

^1^H NMR (500 MHz, CDCl_3_) δ 8.07 (t, *J* = 4.0 Hz, 4H), 7.96–7.88 (m, 4H), 7.86 (s, 2H), 7.83 (s, 2H), 7.79 (d, *J* = 8.5 Hz, 2H), 7.73 (ddd, *J* = 8.3, 5.9, 2.0 Hz, 2H), 7.64 (s, 4H), 7.63–7.59 (m, 2H), 7.38 (ddd, *J* = 8.4, 6.8, 1.3 Hz, 2H), 7.32 (s, 4H), 7.17 (d, *J* = 8.6 Hz, 2H), and 6.64 (s, 2H); ^13^C NMR (126 MHz, CDCl_3_) δ 143.7 (t, *J* = 5.3 Hz), 141.9 (t, *J* = 5.1 Hz), 138.2, 137.9, 133.3, 132.3, 132.2, 132.1, 132.0, 132.0, 131.9, 131.8, 131.8, 131.7, 131.5, 131.4, 131.2, 130.9, 130.7, 129.9, 129.9, 129.5, 129.3, 128.7, 128.0, 127.3, 127.2, 126.8, 126.4, 126.3, 124.3, 124.1, 123.7, 122.1, 122.0, 122.0, 121.9, 121.9, 121.6, 119.9, 119.8, 118.5, 118.3, 118.0, 116.2, 116.0, 115.7, 111.5, and 111.1; ^19^F NMR (470 MHz, CDCl_3_) δ −62.5 (s, 12F), −63.1 (s, 12F), −79.1 (s, 6F), and −117.0 (s, 4F); ^31^P NMR (200 MHz, CDCl_3_) δ −14.7.

### *N*-((11bS)-4-(((11bS)-2,6-bis(3,5-bis(trifluoromethyl)phenyl)-4-(((perfluorophenyl)sulfonyl)imino)-4l5-dinaphtho[2,1-d:1′,2′-f][1,3,2]dioxaphosphepin-4-yl)imino)-2,6-bis(3,5-bis(trifluoromethyl)phenyl)-4l5-dinaphtho[2,1-d:1′,2′-f][1,3,2]dioxaphosphepin-4-yl)-2,3,4,5,6-pentafluorobenzenesulfonamide (IDPi 3) 

^1^H NMR (501 MHz, CD2Cl2) δ = 8.11 (s, 2H), 8.03 (d, *J* = 8.3, 2H), 7.82 (dd, *J* = 3.3, 1.1, 4H), 7.71 (d, *J* = 19.4,4H), 7.67–7.52 (m, 10H), 7.34–7.26 (m, 6H), 7.08 (t, *J* = 7.3, 2H), and 6.60 (s, 2H); ^13^C NMR (126 MHz, CD2Cl2) δ = 143.72, 141.85, 138.34, 137.85, 132.80, 132.20, 131.97, 131.87, 131.81, 131.53,131.42, 131.27, 131.18, 131.15, 130.26, 130.14, 129.98, 129.38, 128.95, 128.65, 128.13, 127.87, 127.14, 127.12, 126.80, 126.59, 124.28, 124.06, 123.53, 122.12, 121.89, 121.71, and 121.33; ^19^F NMR (471 MHz, CD2Cl2) δ = −62.89, −63.43, −137.05 (d, *J* = 20.8), −146.80, and −159.56 to −160.22 (m); ^31^P NMR (203 MHz, CD2Cl2) δ = −15.06.

### *N*-((11bS)-4-(((11bS)-2,6-bis(3,5-bis(trifluoromethyl)phenyl)-4-(((3,5-bis(trifluoromethyl)phenyl)sulfonyl)imino)-4l5-dinaphtho[2,1-d:1′,2′-f][1,3,2]dioxaphosphepin-4-yl)imino)-2,6-bis(3,5-bis(trifluoromethyl)phenyl)-4l5-dinaphtho[2,1-d:1′,2′-f][1,3,2]dioxaphosphepin-4-yl)-3,5-bis(trifluoromethyl)benzenesulfonamide (IDPi 4)

^1^H NMR (400 MHz, CDCl_3_) δ 8.15 (s, 2H), 8.08 (d, *J* = 8.3 Hz, 2H), 7.95–7.82 (m, 10H), 7.74 (s, 2H), 7.69–7.56 (m, 10H), 7.35 (d, *J* = 15.0 Hz, 6H), 7.16 (dd, *J* = 9.8, 7.2 Hz, 4H), and 6.73 (s, 2H); ^13^C NMR (101 MHz, CDCl_3_) δ 144.06, 144.01, 143.96, 142.41, 138.28, 138.16, 132.64, 132.37, 132.26, 132.05, 131.98, 131.92, 131.72, 131.64, 131.59, 131.52, 131.39, 131.26, 131.07, 130.93, 130.57, 130.42, 129.96, 129.72, 129.26, 129.18, 129.12, 128.43, 128.37, 127.88, 127.35, 127.09, 127.04, 126.92, 126.83, 126.71, 126.55, 125.80, 124.64, 124.32, 123.88, 123.81, 121.93, 121.81, 121.61, 121.55, 121.47, 121.09, 119.22, 118.89, and 118.38; ^19^F NMR (376.3 MHz, CDCl_3_): δ −62.56 (s, 4F), −62.91 (s, 4F), and −63.28 (s, 4F); ^31^P NMR (161.9 MHz, CDCl_3_): δ −12.44.

#### 4-((Vinyloxy)methyl)benzaldehyde (scheme S1)

4-(Hydroxymethyl)benzaldehyde (5000 mg, 1 equiv, 36.72 mmol), Na_2_CO_3_ (2.335 g, 0.6 equiv, 22.03 mmol), and a stir bar were added to a 150-ml round-bottom flask with a reflux condenser attached, and is dried under high vacuum. Toluene (50 ml) and vinyl acetate (12.65 g, 13.5 ml, 4 equiv, 146.9 mmol) are added by syringe and stirred for 5 min, after which [IrCl(COD)]_2_ (493.4 mg, 0.02 equiv, 734.5 μmol) dissolved in 10 ml of toluene is added via syringe. The reaction is heated to 90°C, allowed to react overnight, and then cooled to room temperature. The resulting dark brown liquid is diluted with ~30 ml of EtOAc and flushed through a silica plug with 1:1 hexanes:EtOAc to yield a dark brown clear solution. The organic solvent is removed under reduced pressure and the crude product is purified by column chromatography with 20% dichloromethane (DCM) in hexanes on silica to yield a crystalline solid 4-((vinyloxy)methyl)benzaldehyde (4.3 g, 27 mmol, 72%).

^1^H NMR (600 MHz, CDCl_3_) δ 10.04 (s, 1H), 8.01–7.80 (m, 2H), 7.55 (d, *J* = 7.9 Hz, 2H), 6.60 (dd, *J* = 14.3, 6.7 Hz, 1H), 4.88 (s, 2H), 4.33 (dd, *J* = 14.3, 2.4 Hz, 1H), and 4.16 (dd, *J* = 6.7, 2.4 Hz, 1H); ^13^C NMR (151 MHz, CDCl_3_) δ 191.90, 151.31, 143.86, 135.92, 129.98, 127.53, 87.93, and 69.15; HR-MS (ESI+)(C_10_H_10_O_2_)[M + MeOH+Na]+: expected 217.08406, found: 217.0846; FT-IR (powder) νmax/cm^−1^: 3125, 2928, 2840, 2744, 1689, 1608, 1323, 991, 821, 574, and 434.

### 6-Oxo-1,5-diphenyl-3-(4-((vinyloxy)methyl)phenyl)-5,6-dihydro-1*H*-1,2,4,5-tetrazin-2-yl

2,4-Diphenyl-6-(4-((vinyloxy)methyl)phenyl)-1,2,4,5-tetrazinan-3-one (1000 mg, 1 equiv, 2.588 mmol; scheme S2) was suspended in DCM (20 ml) in a 150 ml thick-walled pressure tube, and p-benzoquinone (0.5 g, 1.7 equiv, 4.39 mmol) was added to the reaction, upon which the color changed to a light yellow-orange. The reaction was sealed and heated to 60°C while stirring. After 3 hours, the heat source was removed and the reaction allowed to cool to room temperature, then the crude reaction mixture was washed with 100 ml of deionized water and 4× 100 ml of 1 M NaOH (until aqueous layer is clear). Three spots were observed by TLC analysis (20% Et_2_O/pentane), with the product being the first red spot at Rf ≈ 0.40. The organic layer is collected, concentrated under vacuum, and dry loading onto 300 wt % celite. Purification of the material was accomplished with flash chromatography (10 to 20% Et_2_O in hexanes) on silica gel to afford pure 6-oxo-1,5-diphenyl-3-(4-((vinyloxy)methyl)phenyl)-5,6-dihydro-1*H*-1,2,4,5-tetrazin-2-yl (803 mg, 2.09 mmol, 80.9%) and fluffy crystalline red solid.

EPR (CHCl_3_): 2.0044 (4 N); FT-IR (powder, cm^−1^): 3056, 1696, 1650, 1486, 1318, 1203, 1177, 997, 742, 560; HR-MS (ESI+)(C_23_H_19_N_4_O_2_*Na) [M + Na] + Expected:406.1405 Found: 406.1388; XRD: CCDC # 2324183.

Significant streaking occurs at mass loadings on column >1 wt%. NaOH (1 M) washes are crucial for removing benzoquinone adduct with product.

### Polymerization of oxoverdazyl benzyl vinyl ether (scheme S3)

6-Oxo-1,5-diphenyl-3-(4-((vinyloxy)methyl)phenyl)-5,6-dihydro-1*H*-1,2,4,5-tetrazin-2-yl (120 mg, 1 equiv, 313 μmol), 3.6 ml of PhMe, and a stir bar were added to a 2-g vial under an inert atmosphere and stringently dry conditions. Separately, a solution of IDPi (1 mol %, 3.13 μmol) in 0.4 ml of PhMe was prepared and charged to a separate vial. The vial containing the monomer solution and stir bar was cooled to −78°C, after which the solution of catalyst was added. The reaction was allowed to stir at temperature for 2 hours before being quenched with 5% Et_3_N in MeOH and allowed to warm to room temperature. Hexanes are added to fully precipitate the polymer, which is then filtered under vacuum through a Nylon 66 membrane twice (normal filter paper works also but tends to stick). The polymer is then washed with copious amounts of MeOH to yield pure product.

Note that the monomer solution in toluene is purple/pink but turns to a deep red upon cooling to −78°C. As the reaction progresses the solution turns back to the original monomer color as the polymer partially precipitates. We observed that if no reactivity occurs due to impurities, polymer can still be produced by warming to −40°C; however, a loss of selectivity occurs.

FT-IR (cm^−1^, powder) 3091, 2922, 1694, 1591, 1483, 1358, 1119, 823, 746, and 507; EPR (CH_2_Cl_2_, 1 mg/ml): sigmoidal, *g* = 2.003; *T*_d/5 wt%_: 246°C, 10°/min; *T*_g, midpoint_: 60°–70°C (weak); *T*_m, peak_: 140°–150°C (tacticity dependent, can only be seen on first heating cycle as cross-linking occurs after melt). See the Supplementary Materials for polymer reduction and subsequent NMR analysis.

## Supplementary Materials

### This PDF file includes:

Supplementary Text Schemes S1 to S3 Figs. S1 to S31 Tables S1 to S7 Data S1 and S2 References
