# Supplementary material for: Stereoregular radical polymers enable selective spin transfer
Source: Sci Adv. 2025 Mar 21;11(12):eadr4004. doi: 10.1126/sciadv.adr4004 (PMC11927620; doi:10.1126/sciadv.adr4004)
Supplement: Supplementary file 1 — Supplementary Text Schemes S1 to S3 Figs. S1 to S31 Tables S1 to S7 Data S1 and S2 References [file sciadv.adr4004_sm.pdf]

Science Advances
AAAS

Supplementary Materials for

Stereoregular radical polymers enable selective spin transfer

Hyunki Yeo *et al.*

Corresponding author: Frank A. Leibfarth, frankl@email.unc.edu; Bryan W. Boudouris, boudouris@purdue.edu; Brett M. Savoie, bsavoie@purdue.edu

Sci. Adv. **11**, eadr4004 (2025)
DOI: 10.1126/sciadv.adr4004

This PDF file includes:

Supplementary Text
Schemes S1 to S3
Figs. S1 to S31
Tables S1 to S7
Data S1 and S2
References

Major Structures and Schemes Utilized in Study

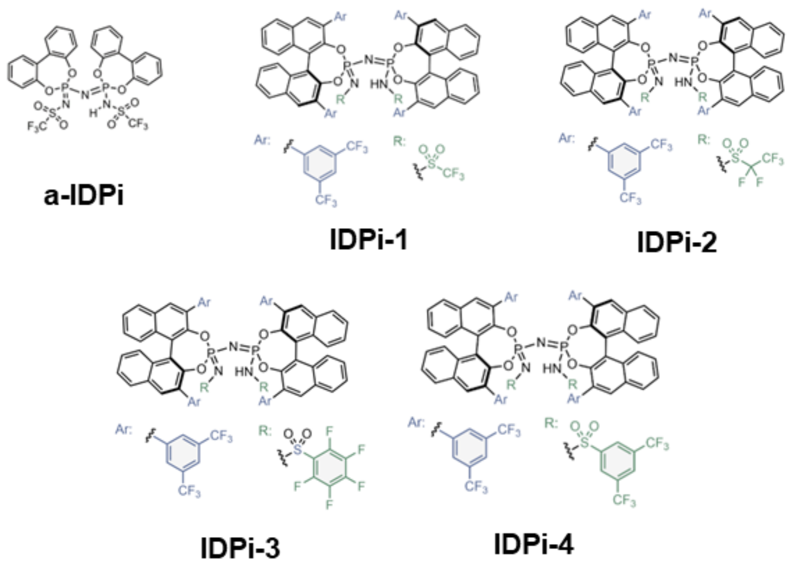

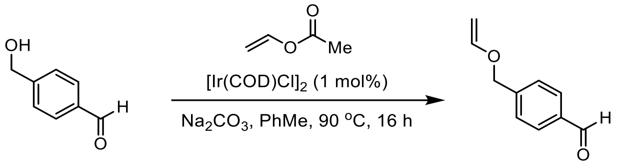

Scheme S1. Synthesis of 4-((vinyloxy)methyl)benzaldehyde

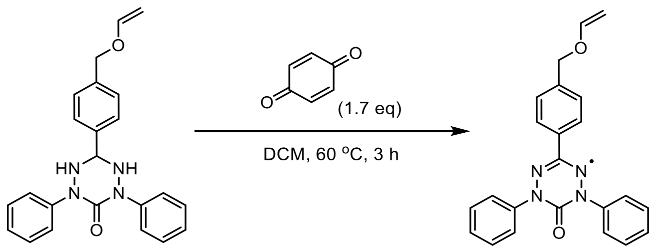

Scheme S2. Synthesis of 6-oxo-1,5-diphenyl-3-(4-((vinyloxy)methyl)phenyl)-5,6-dihydro-1H-1,2,4,5-tetrazin-2-yl. 2,4-diphenyl-6-(4-((vinyloxy)methyl)phenyl)-1,2,4,5-tetrazinan-3-one

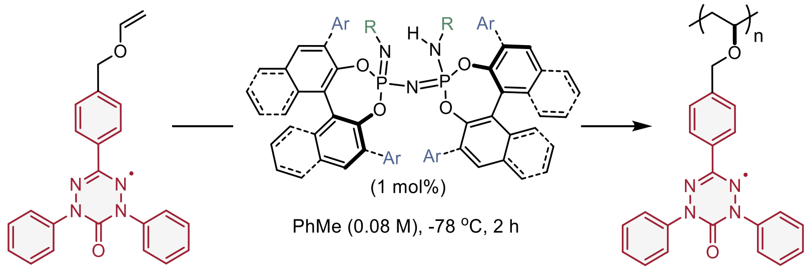

Scheme S3. Polymerization of Oxoverdazyl Benzyl Vinyl Ether

1. Molecular Analysis of Synthesized Materials

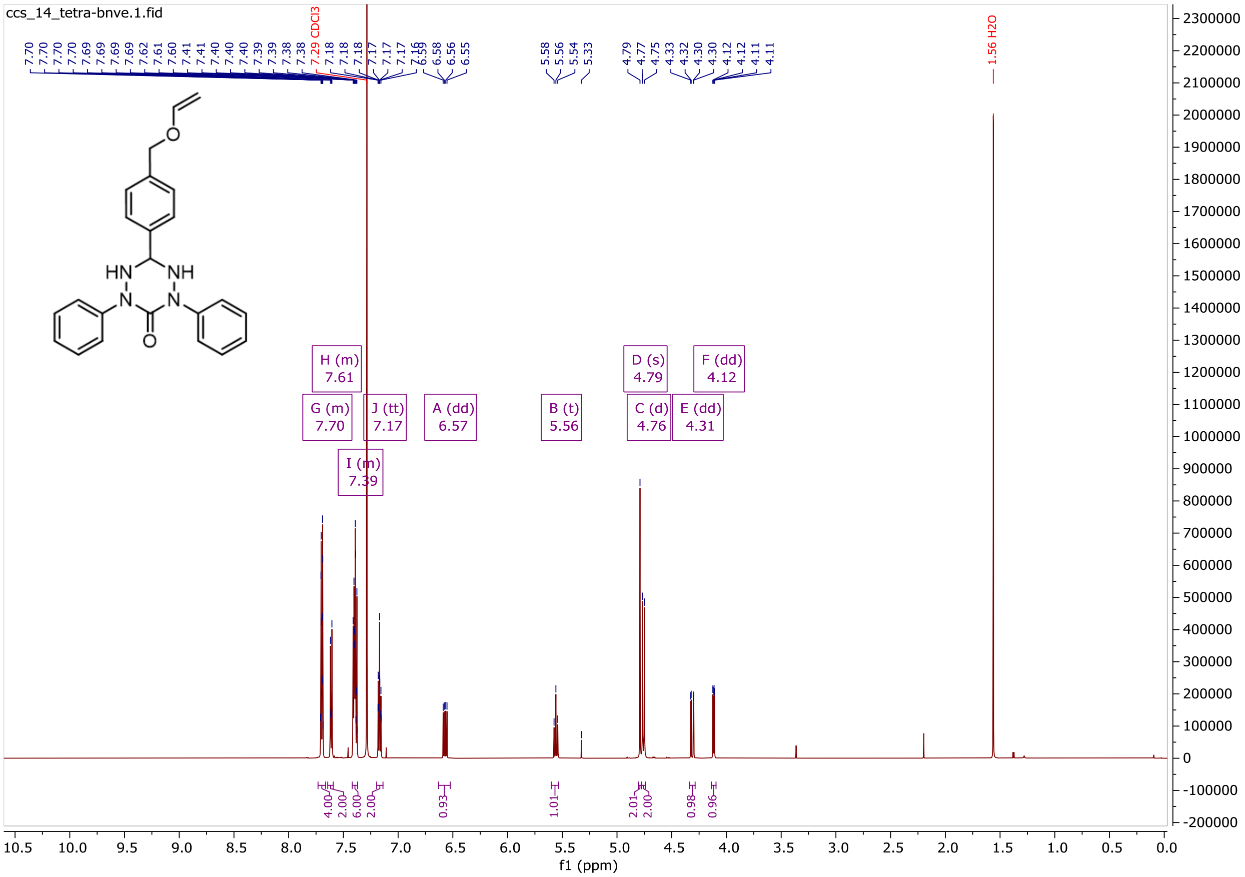

Fig. S1: ^{1}H NMR (600 MHz, $CDCl_3$) of 2,4-diphenyl-6-(4-((vinyloxy)methyl)phenyl)-1,2,4,5-tetrazinan-3-one.

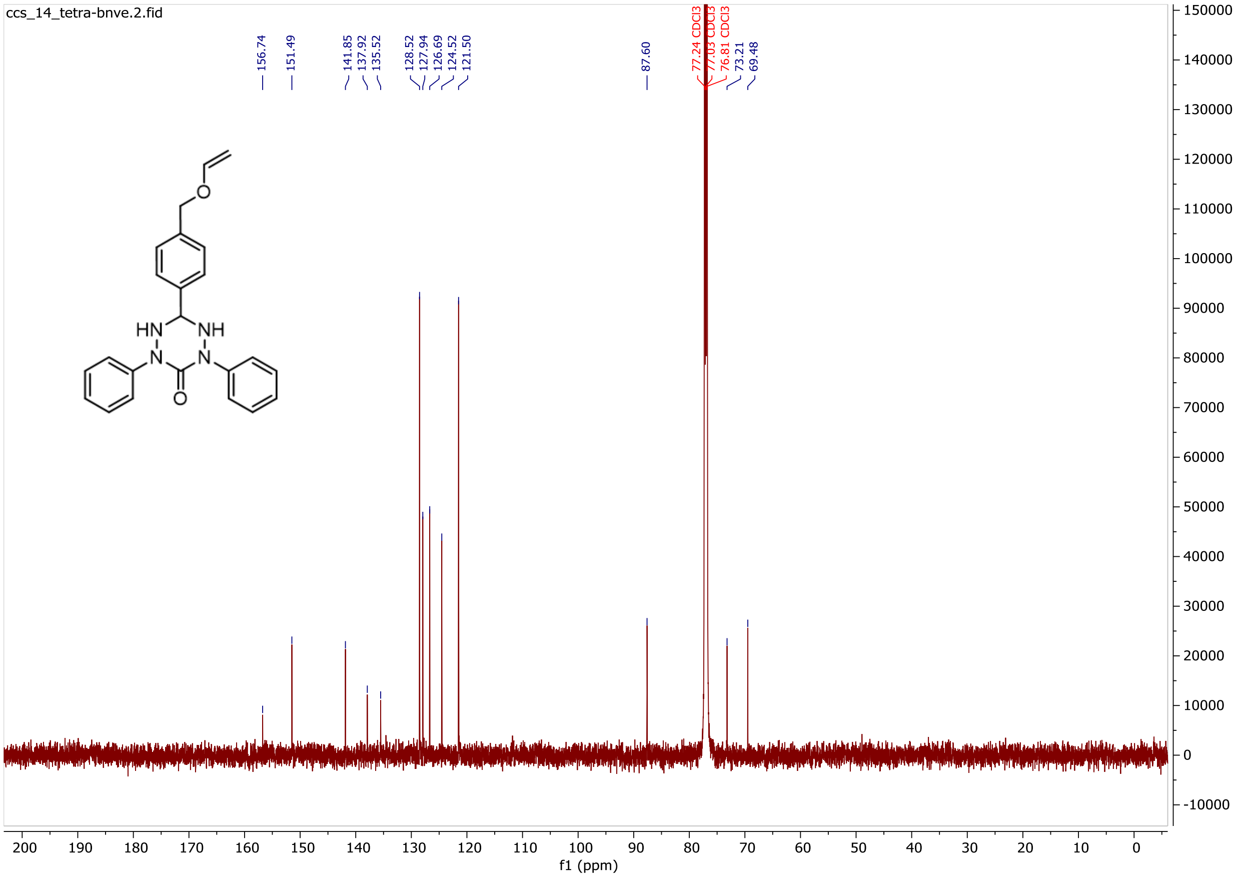

Fig. S2: ^{13}C NMR (151 MHz, $CDCl_3$) of 2,4-diphenyl-6-(4-((vinyloxy)methyl)phenyl)-1,2,4,5-tetrazinan-3-one.

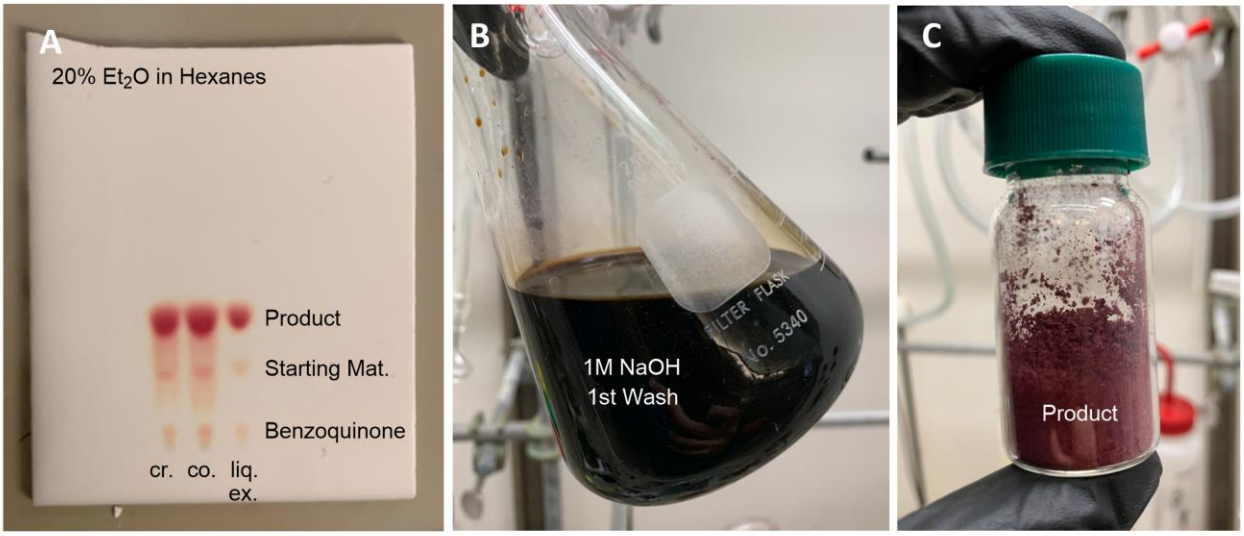

Fig. S3: Photos of reactants and product. (A) TLC place of crude material, co-spot, and crude material after liquid extraction with 1M NaOH, (B) aqueous 1M NaOH phase extraction, and (C) purified oxo-verdazyl monomer.

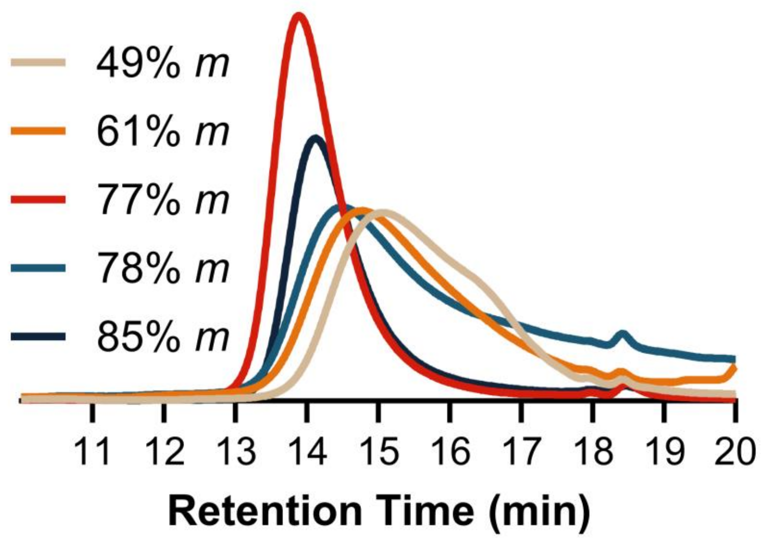

Fig. S4: Gel permeation chromatography of radical containing polymer in $CHCl_3$ at 1 mg/ml.

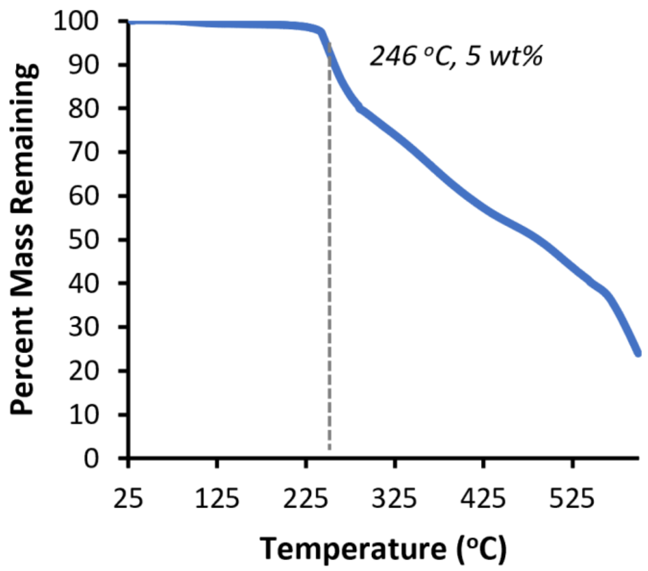

Fig. S5: Gravimetric thermogram of P1-49% ***m (representative trace)*** **from room temperature to 600 °C.**

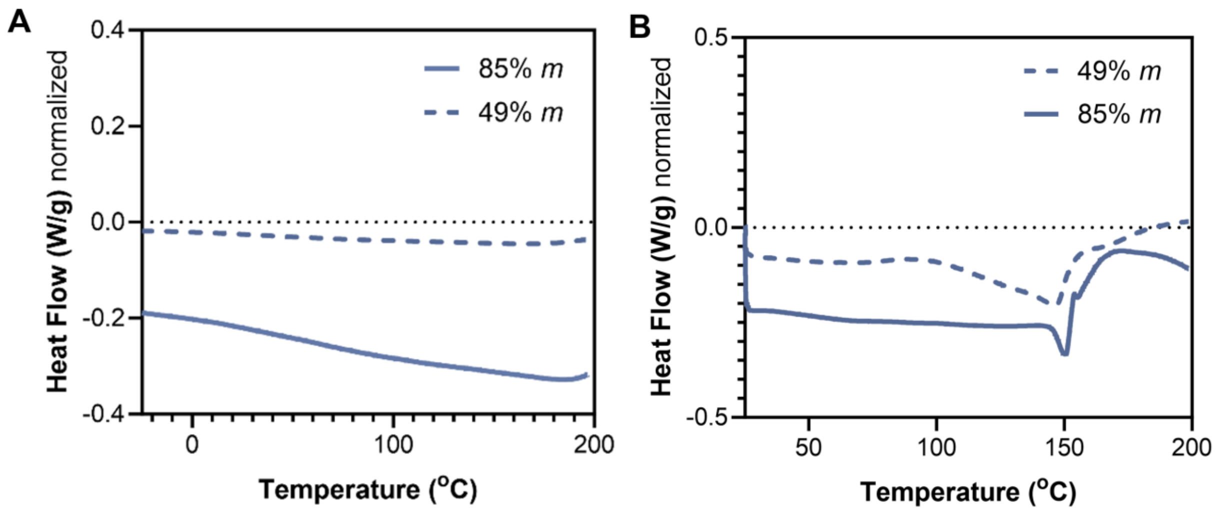

Fig. S6: **Differential scanning calorimetry of P1 49% *m* and 85% *m* (scan rate of 10 ºC/min). A**) second heating cycle and **B**) first heating cycle where material appears to crosslink.

Table S1: Tabulated polymerizations of material characterized in this study.

Entry	Monomer	Catalyst	Conditions	Mass (mg)	Yield *(iso)*	% *m*	M_n (RI)
**1**	**M1**	**α-IDPi**	PhMe (0.08 M), -78 C, 2 hr	112	93%	**49**	3.8
**2**	**M1**	**IDPi 1**	PhMe (0.08 M), -78 C, 2 hr	108	90%	**78**	4.7
**3**	**M1**	**IDPi 3**	PhMe (0.08 M), -40 C, 2 hr	32	64%	**71**	--
**4**	**M1**	**IDPi 2**	PhMe (0.08 M), -40 C, 2 hr	105	86%	**61**	5.7
**5**	**M1**	**IDPi 2**	PhMe (0.08 M), -78 C, 2 hr	94	78%	**85**	11.9
**6**	**M1**	**IDPi 3**	PhMe (0.08 M), -78 C, 2 hr	103	86%	**77**	15.8
**7**	**M2**	**IDPi 4**	PhMe (0.08 M), -78 C, 2 hr	0	--	--	--

Reduced Poly(oxo-verdazyl benzyl vinyl ether)

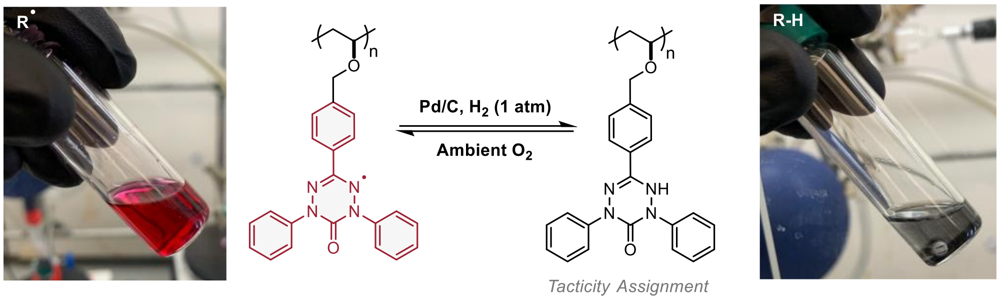

**Radical solution on left is diluted to show color, typically a deep opaque red.*

For tacticity assignment and assurance of purity, the radical form of poly(oxo-verdazyl benzyl vinyl ether) was reduced according this procedure:

A sample of radical polymer (30-50 mg) was charged to a 2-dram vial containing a stir bar with 10 wt%/w of Pd/C (10 wt%) and cycled three times with N_2 and vacuum. To this vial was added d^4-tetrachloroethane (0.7 mL) and three drops of d^4-MeOH, which fully dissolved the polymer sample. A H_2 balloon was added via syringe and allowed to react for 1-2 hours (or until the reaction turns colorless, i.e., no red color).

After completion, the balloon was removed from the reaction, quickly taped over, and transferred into a glove box, where the solution was filtered through a 0.2-micron PTFE filter into a pre-dried J-young NMR tube for NMR analysis.

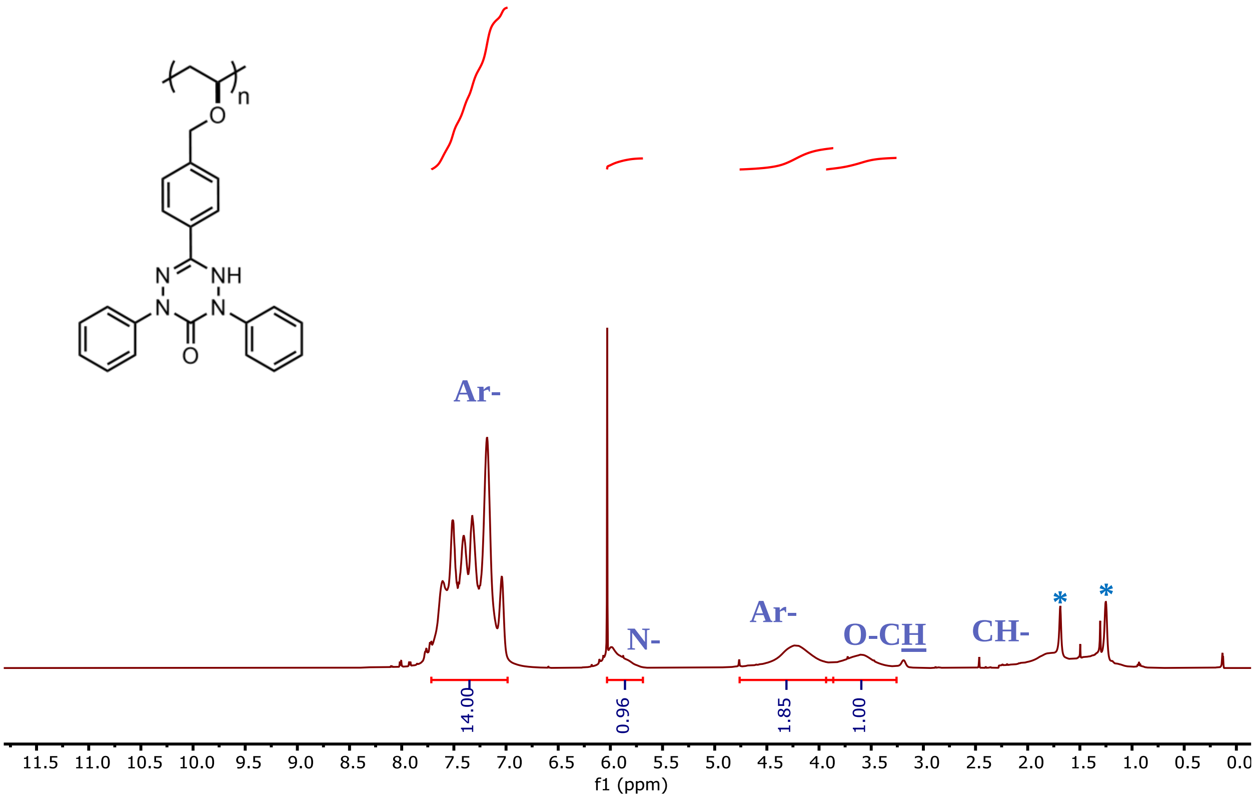

Fig. S7: **^{1}H NMR (600 MHz, $C_2D_2Cl_4$) sample of the reduced leuco-verdazyl form of poly(oxo-verdazyl benzyl vinyl ether),** 78% *m*. *MeOH, H_2O impurities. Methylene protons are convoluted between 2.0-1.0 ppm.

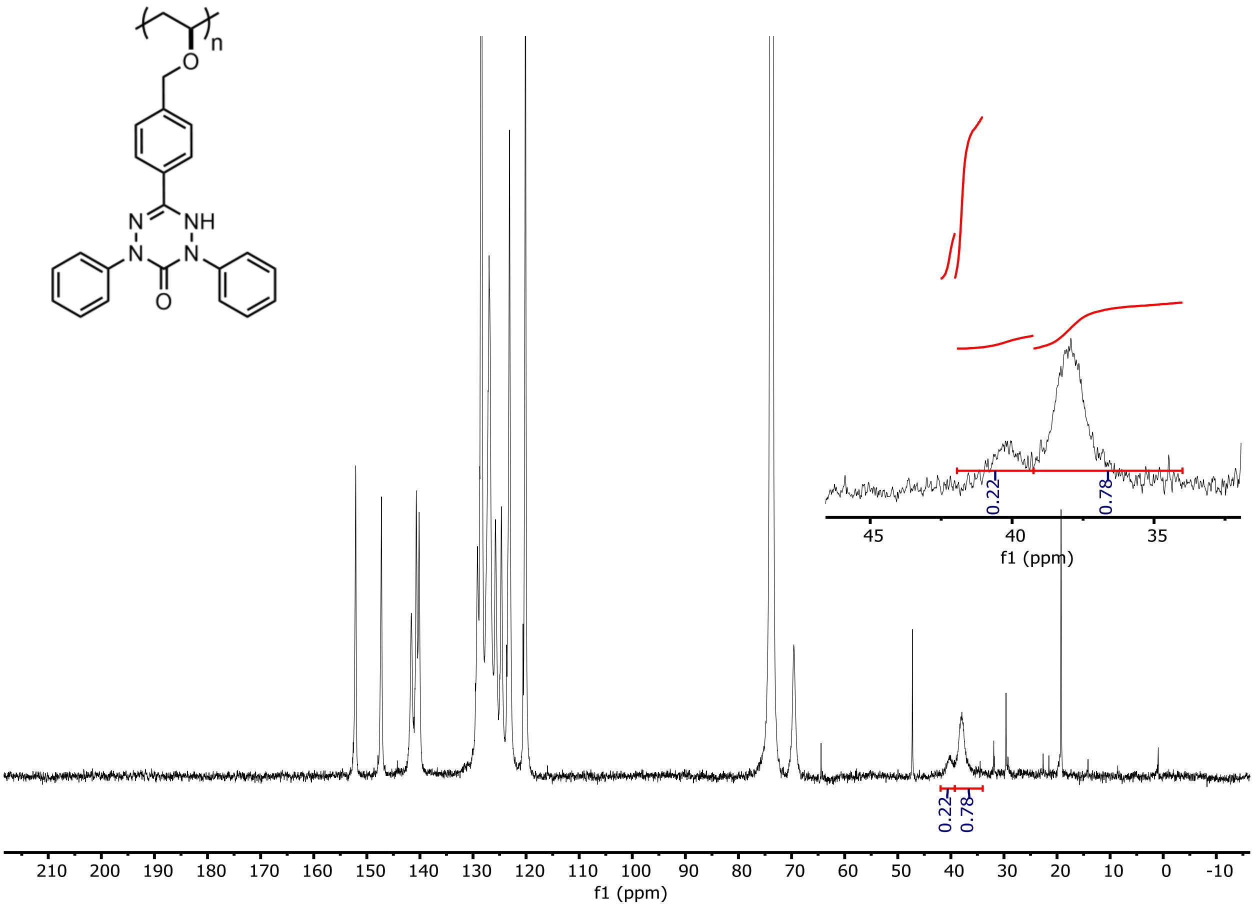

Fig. S8: **^{13}C NMR (600 MHz, $C_2D_2Cl_4$) sample of the reduced leuco-verdazyl form of poly(oxo-verdazyl benzyl vinyl ether),** 78% *m*. *MeOH, H_2O impurities.

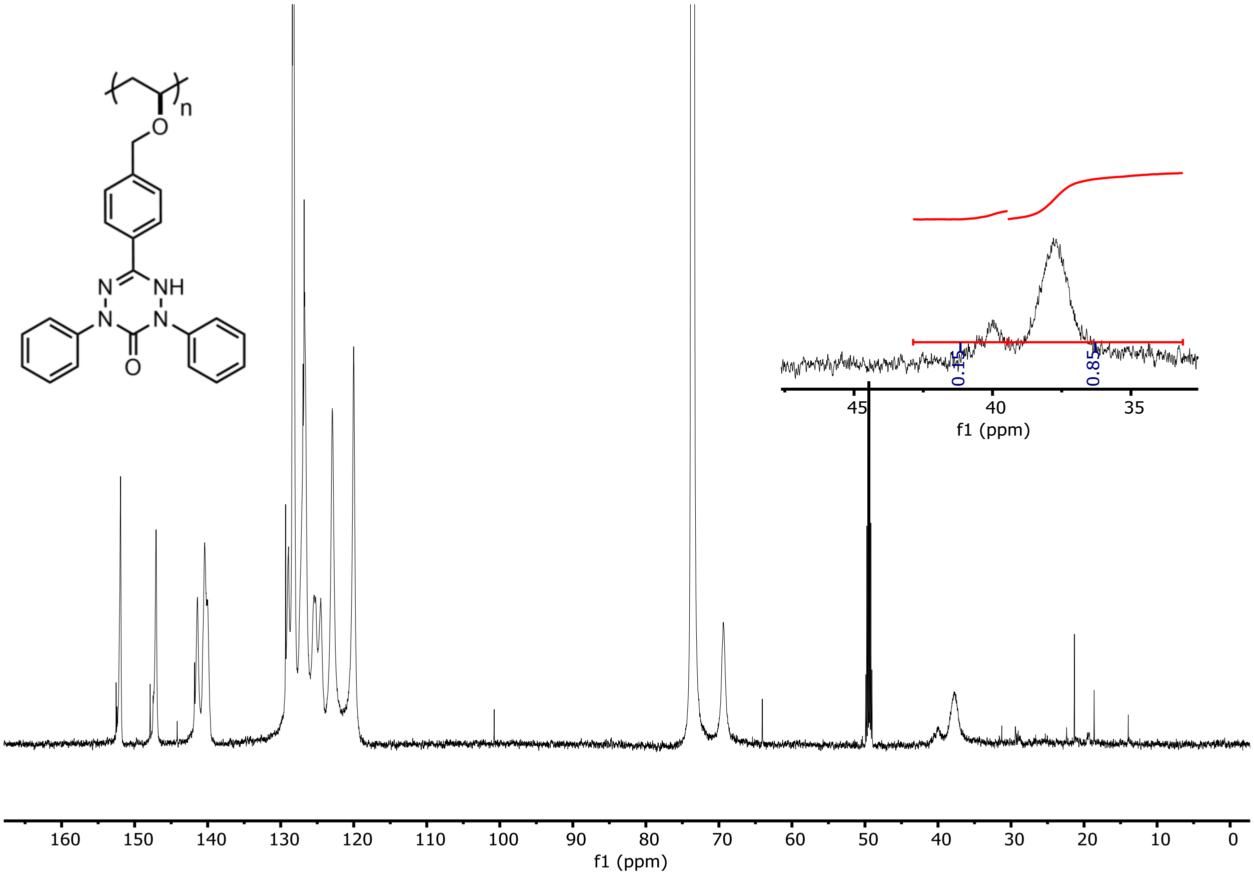

Fig. S9: ^{13}C NMR (600 MHz, $C_2D_2Cl_4$) sample of the reduced leuco-verdazyl form of poly(oxo-verdazyl benzyl vinyl ether), 85% *m*. *d^4-MeOH, MeOH, H_2O impurities.

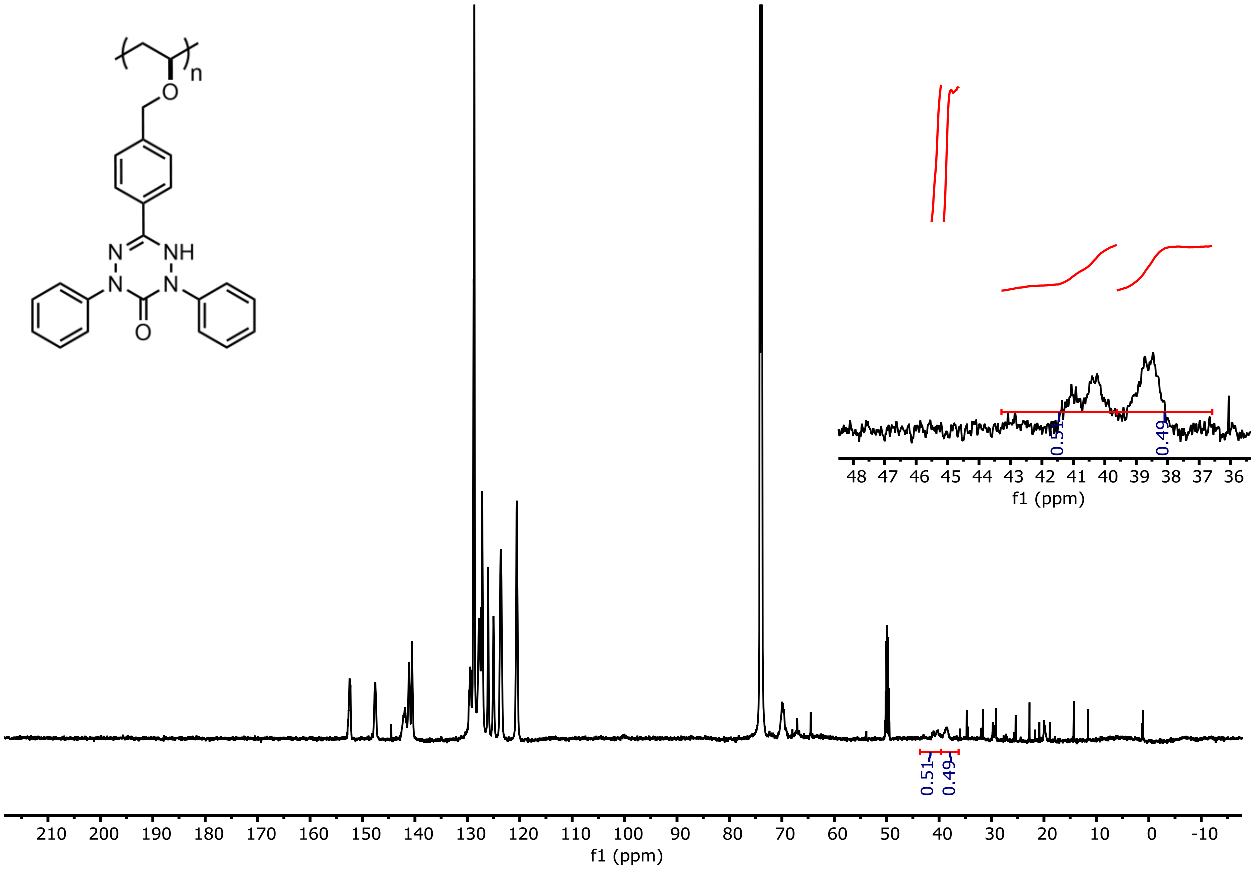

Fig. S10: ^{13}C NMR (600 MHz, $C_2D_2Cl_4$) sample of the reduced leuco-verdazyl form of poly(oxo-verdazyl benzyl vinyl ether), 49% *m*. *d^4-MeOH, MeOH, H_2O impurities. *Note: atactic material showed some degradation during the reduction process (0-35 ppm).*

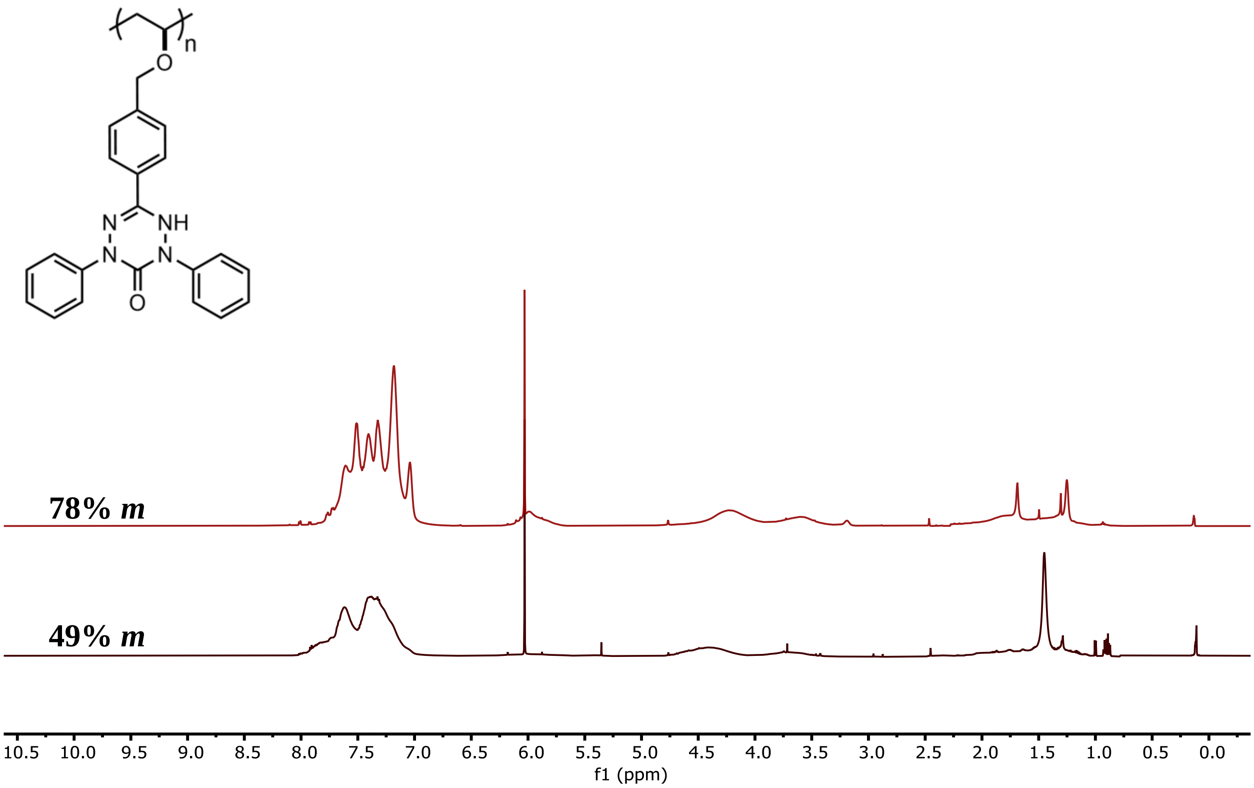

Fig. S11: ^{1}H NMR (600 MHz, $C_2D_2Cl_4$) sample of the reduced leuco-verdazyl form of poly(oxo-verdazyl benzyl vinyl ether), 49% and 78% *m*. *MeOH, H_2O impurities. Methylene protons are convoluted between 2.0-1.0 ppm.

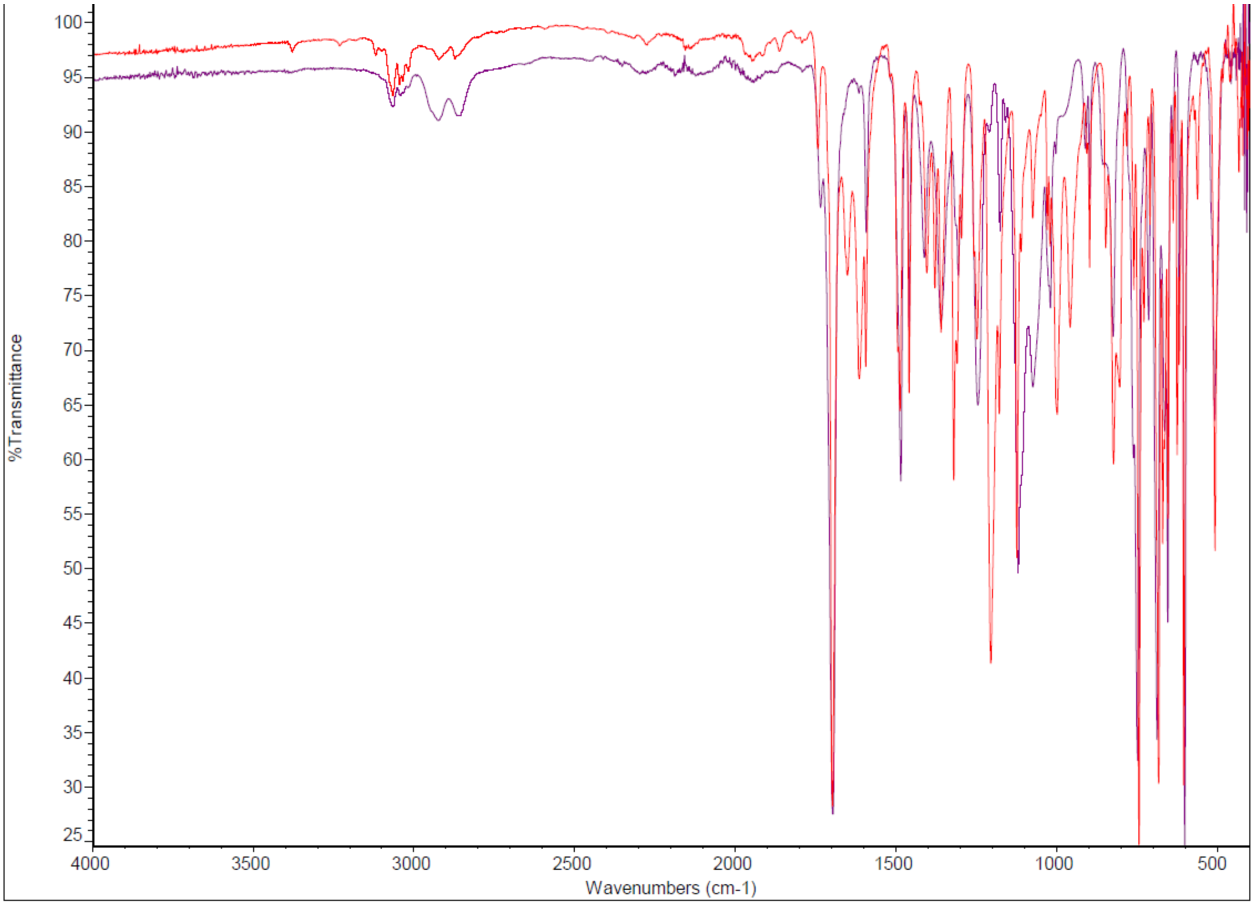

Fig. S12: FT-IR spectra (neat) of the monomer (red) and resulting polymer (purple). Consumption of vinyl ether is observed by loss of peak around 1650 cm^{-1-} and generation of aliphatic stretches <3000 cm^{-1}.

2. Supplementary Text

2.1 Single Crystal Junction Device Fabrication

A nicely grown sheet like M1 single crystal was picked from a vial *(see X-ray crystallography section for growth condition*), spun-coated with several drops of 30: 70 wt % ethanol: chloroform solution to stick on a silicon wafer surface. After covering the immobilized single crystal with a silver single wire mask (d: 110 µm), the silicon wafer was deposited in a thermal evaporator installed in inert atmosphere glovebox. Then, 200 nm of silver was deposited on top of the films at a reduced pressure of ~10^{-6} bar. After deposition, the silver wire mask was removed. Optical images were taken to measure the dimensions of the single crystal, and further magnetoelectric tests for demonstrated in a magnetic probe station connected to a in reduced pressure of ~10^{-7} bar.

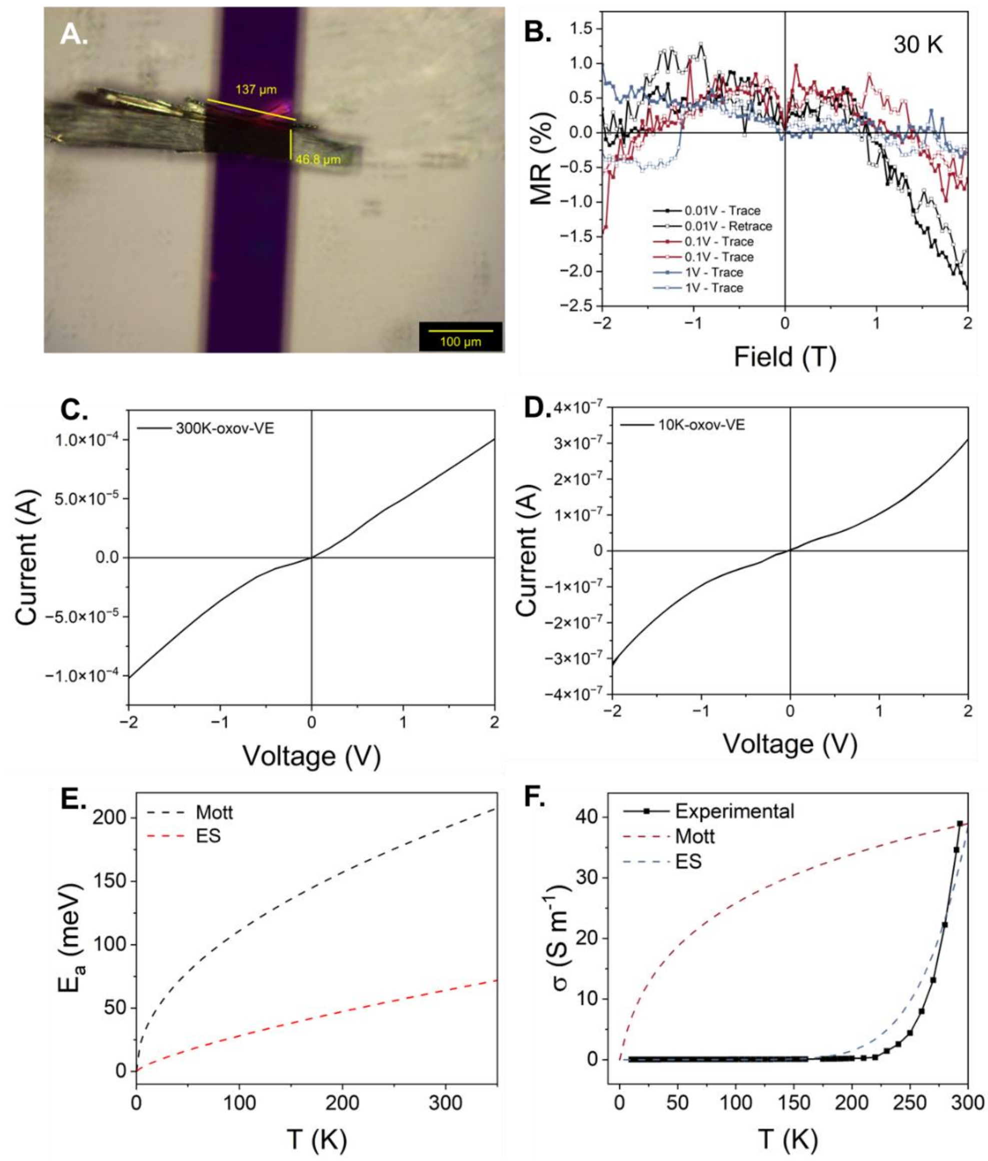

Fig. S13. Charge transport properties of single crystal SC junction device made of M1. (A) Top- view image took from a polarized optical microscope. (B) MR profiles of SC junction device at 30K. Represented I-V curves of the SC junction device at (C) 300K and (D) 10K. (E) Activation energy fitting based on ES and Mott VRH models. (F) σ vs T plots of ES and Mott models based on fittings calculated from (E).

2.2 Alternative Cationic Polymerization Catalysts

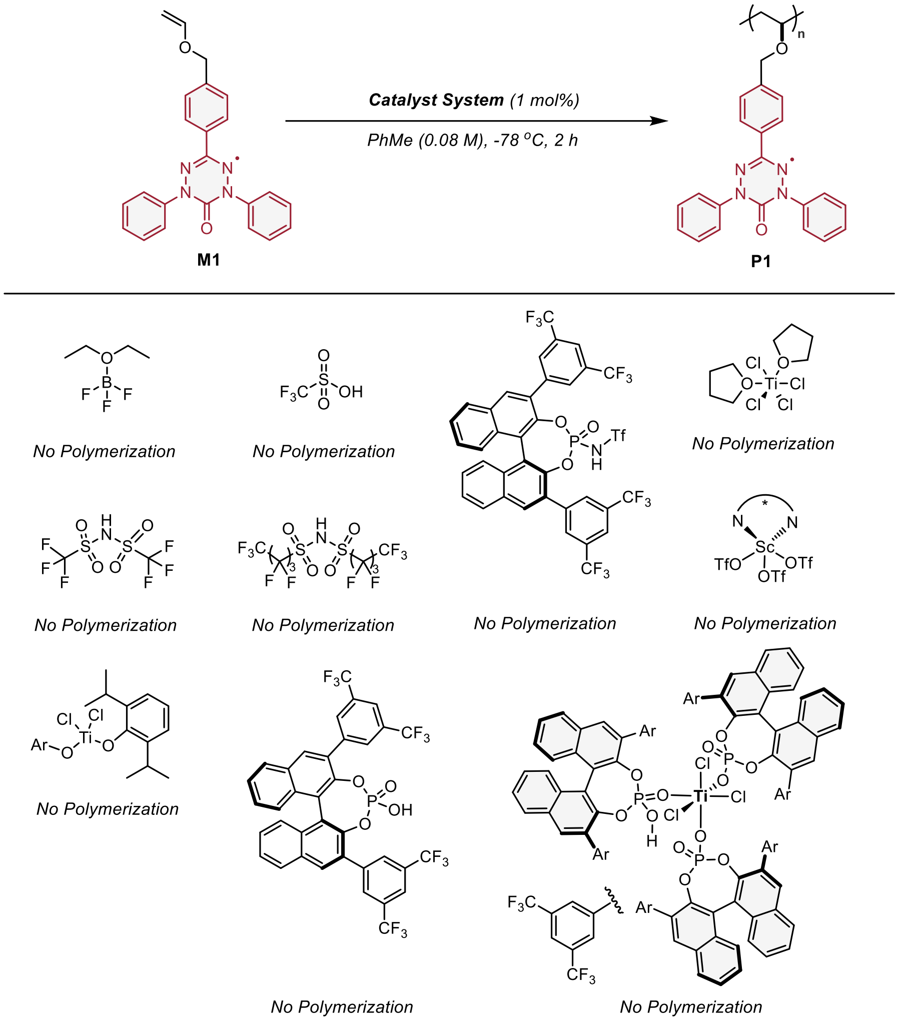

Fig. S14. Other catalysts/initiators tested for the cationic polymerization of M1, showcasing the utility of IDPis.

2.3 Magnetism Measurements

The magnetic susceptibility of **P1** were measured in a Quantum Design superconducting quantum interference device (SQUID) magnetometer. The samples were prepared by placing ~2 mg of the material of interest in a clean, dry polycarbonate capsule. Vibrating sample mode (VSM) was utilized for all the measurements. The sample was zero-field cooled to 2 K, then the magnetic susceptibility was measured from 2 to 300 K in intervals of 0.03 K.

The magnetization of **P1** was measured using the same SQUID magnetometer. A field of -7 T $\leq$ B $\leq$ +7 T was applied to the sample and the moment was measured at T = 2 K, 2.5K, 3 K, 5 K, 10 K, 20 K, 50 K, 100 K, 200 K and 300 K. The magnetic susceptibility of **P1** were corrected for the contribution of the diamagnetic susceptibility. Using Pascal's constants, the diamagnetic susceptibility of **P1** was -5.03×10^{-6} emu mol^{-1}. The molar magnetic susceptibility was then fit to the Curie-Weiss law, as described by Equation 1.

$$\chi_m = \frac{C}{T-\theta} \qquad \textbf{(Equation S1)}$$

Here, C is the Curie constant, and θ is the Curie temperature. The Curie constants of **P1** and molar magnetic susceptibility χ_m at 2K and 300K are indicated in Table S1. These values are comparable to theoretical values of χ_m at 300 K for an unpaired electron as 1.25×10^{-3} emu mol^{-1}. The similarity of measured and theoretical magnetic susceptiblity is due to the high radical content of **P1**, consistent with the EPR data shown above.

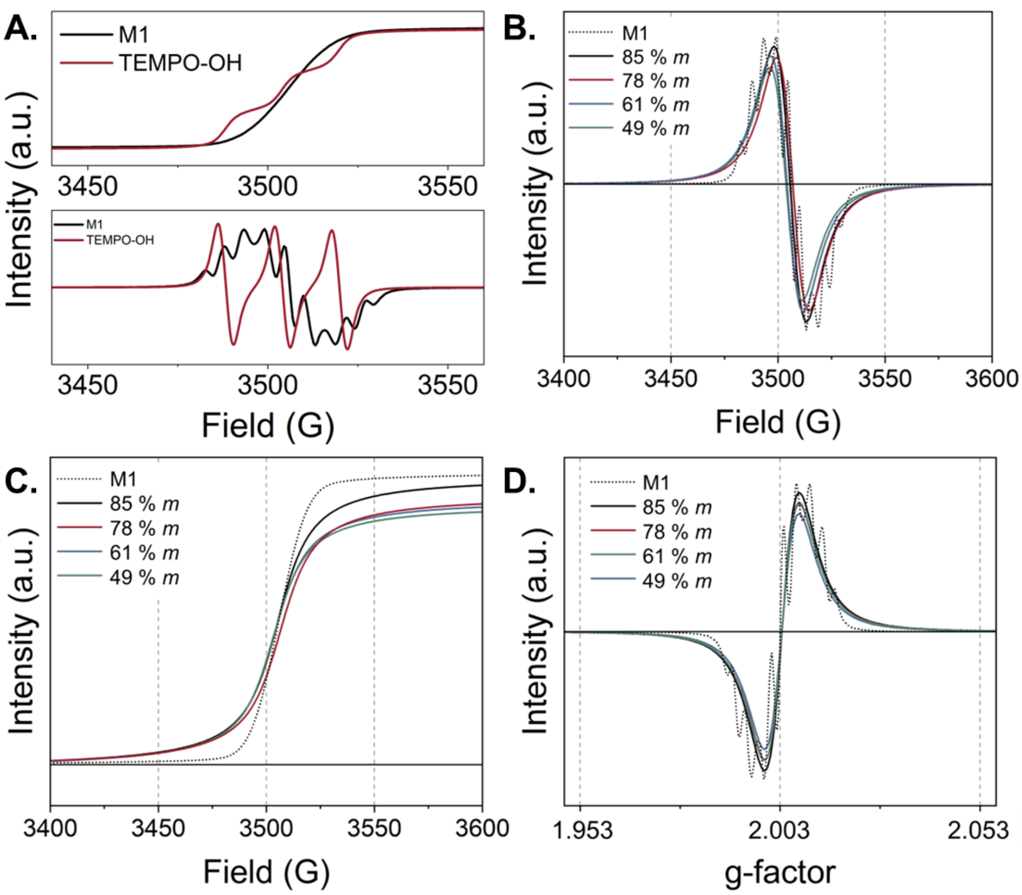

Fig. S15. EPR spectroscopy of following materials. (A) monomer M1 compared with TEMPO-OH standard sample, (B) P1 depending on tacticity and (C) double integration of EPR signal to calculate radical content. All polymers performed > 95 % of radical content with a Lorentzian curve compared to its monomer signal, indicating that the synthesis did not terminate radicals and the resulting polymer has intramolecular spin-spin interaction. (D) g-factors of all samples were determined to be ~ 2.003.

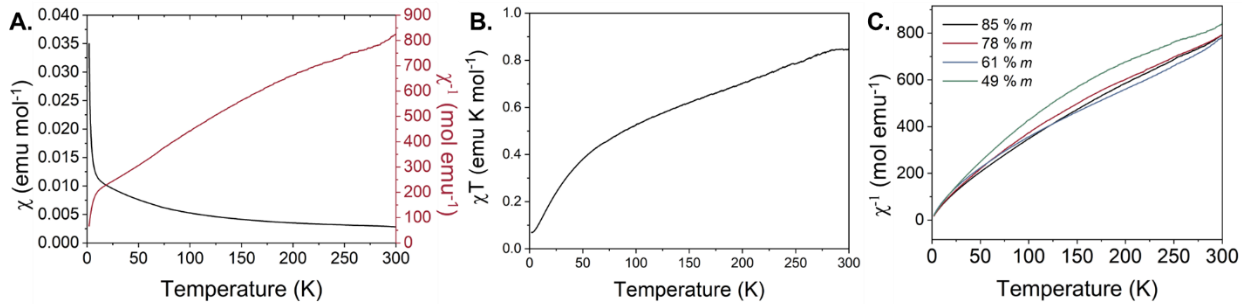

Fig. S15. Bulk magnetic properties measured by SQUID. (A) χ vs T and χ-1 vs T plots of monomer M1 obtained from SQUID spectrometer. (B) χT vs T plot of monomer M1. (C) χ-1 vs T plots of P1 obtained from SQUID spectrometer. In general, all P1 show paramagnetic behavior.

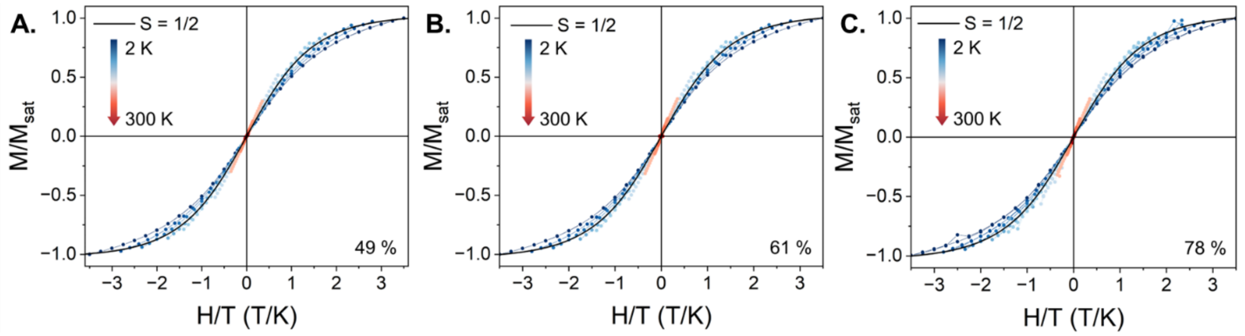

Fig. S16. Magnetism plots of P1 in and order of (A) 49 %, (B) 61 % and (C) 78 % m. All four polymers follow the Brillouin function models of S = 1/2.

Table S2. Calculated parameters of the Curie-Weiss fit from the experimental results collected from SQUID spectroscopy.

**Material**	$\chi_{m,2K}$ **(emu mol^{-1})**	$\chi_{m,300K}$ **(emu mol^{-1})**	**C (emu)**	**θ (K)**
M1	0.03451	1.23×10^{-3}	8.14	9.14
P1 – 85 % m	0.05791	1.27×10^{-3}	1.96	- 0.868
P1 – 78 % m	0.0566	1.26×10^{-3}	2.13	- 0.843
P1 – 61 % m	0.04865	1.28×10^{-3}	1.97	- 0.885
P1 – 49 % m	0.05146	1.19×10^{-3}	2.25	- 0.899

When a high field is applied to a paramagnet, all the spins of the system align to the field and the measured magnetization asymptotes to a value of M_{sat}. The magnetization behavior of the paramagnetic system can then be modeled using a Brillouin function, $B_s(x)$, where the following equations hold.

$$M = M_{sat} B_s(x) \qquad \textbf{(Equation S2)}$$

Here,

$$B_s(x) = \frac{2S+1}{2S} coth\left(\frac{2S+1}{2S} \cdot x\right) - \frac{1}{2S} coth\left(\frac{1}{2S} \cdot x\right) \qquad \textbf{(Equation S3)}$$

and

$$x = \frac{gS\mu_B H}{k_B T} \qquad \textbf{(Equation S4)}$$

where g is the g-factor and S is the electron spin quantum number. The Brillouin function models the behavior of a paramagnet without any interactions between the spins. Because the verdazyl radical has a single unpaired electron, the measured magnetization curves fit the Brillouin curve for an S value of 0.5 (Fig. S20).

2.4 Spin Valve Device Fabrication and Measurements

8.3 mm × 8.3 mm glass substrates were cleaned in acetone, chloroform, and isopropyl alcohol by sonication for 10 min each in a sequential order. The substrates were then placed in a thermal evaporator installed in inert atmosphere glovebox. Then, 10 nm of lanthanum strontium manganite (LMSO) was deposited on top of the films at a reduced pressure of ~10^{-6} bar to yield a I-shape electrode area. **P1** was dissolved in chloroform at a concentration of 50 mg mL^{-1}. In air, the solutions were spin-coated on the ITO substrates at a rate of 2,000 rpm for 30 s to form a layer that was ~100 nm thick, as measured using a Bruker Dektak XT stylus profilometer. The films were annealed at 50 °C for 10 min to remove the residual solvent. The films were then placed in a thermal evaporator installed in inert atmosphere glovebox, with the same mask used for LMSO deposition but faced perpendicularly to the original pattern. Then, 20 nm of cobalt (20nm) was deposited on top of the films at a reduced pressure of ~10^{-6} bar. The cross-sectional device active area where the upper LMSO and lower Co layer overlaps was calculated as 1.357 mm^2. Initial room-temperature conductivity values were calculated by using a PS100 Lakeshore probe station with a Keithley 2400 source meter to control the voltage values in the range of $-2\text{ V} \leq V \leq +2\text{ V}$.

Fabricated devices were mounted on a universal board, 4-point wired and installed to a Quantum Design DynaCool PPMS. The resistance (R) values were determined by sweeping voltage and measuring current (I-V curves) and then taking the slopes of voltage versus current plot as the corresponding resistance base on Ohm's law (Fig. S). All I-V curves were linear. The electrical conductivity of the sample was determined using the device channel length (L) and cross-sectional area (A), as characterized using optical microscopy with the following equation.

$$\sigma = \frac{L}{RA} \qquad \textbf{(Equation 5)}$$

The validity of this assumption was double checked with a Bruker Dektak XT stylus profilometer. Then, collected datasets were plotted fitted to the following equation to obtain parameters σ_0 and T_0 which do not have any physical meaning, by implying two different types of variable range hopping (VRH) models Mott ($\rho = 4$) and ES ($\rho = 2$), respectively.

$$\sigma = \sigma_0 e^{-(T_0/T)^{1/p}} \qquad \textbf{(Equation S5)}$$

Activation energy (E_a) was plotted by following the Equation S6 for Mott's model and Equation S7 for ES model, where k is Boltzmann's constant. Calculated parameters are noted on Table S3.

$$E_a = 0.125kT_0^{1/4}T^{3/4} \qquad \textbf{(Equation S6)}$$

$$E_a = 4.579kT_0^{1/2}T^{1/2} \qquad \textbf{(Equation S7)}$$

To calculate magnetoresistance (MR), bias voltage was applied by measuring the current in different magnetic fields. OMAR of SC junctions were measured while leaving the current direction perpendicular to the field, however, spin valve GMR studies were demonstrated after rotating the plane parallel to the field. In general, MR can be expressed as the following equation,

$$MR = 100 \cdot \frac{\Delta R}{R_0} (\%) = 100 \cdot \frac{R_B - R_0}{R_0} \qquad \textbf{(Equation S8)}$$

where R_B stands for the resistance value in a field of B and R_0 stands for the resistance at zero field. Field sweeps were held in various temperatures and voltages values per device with a field relaxation time of 100 ms (NPLC = 5).

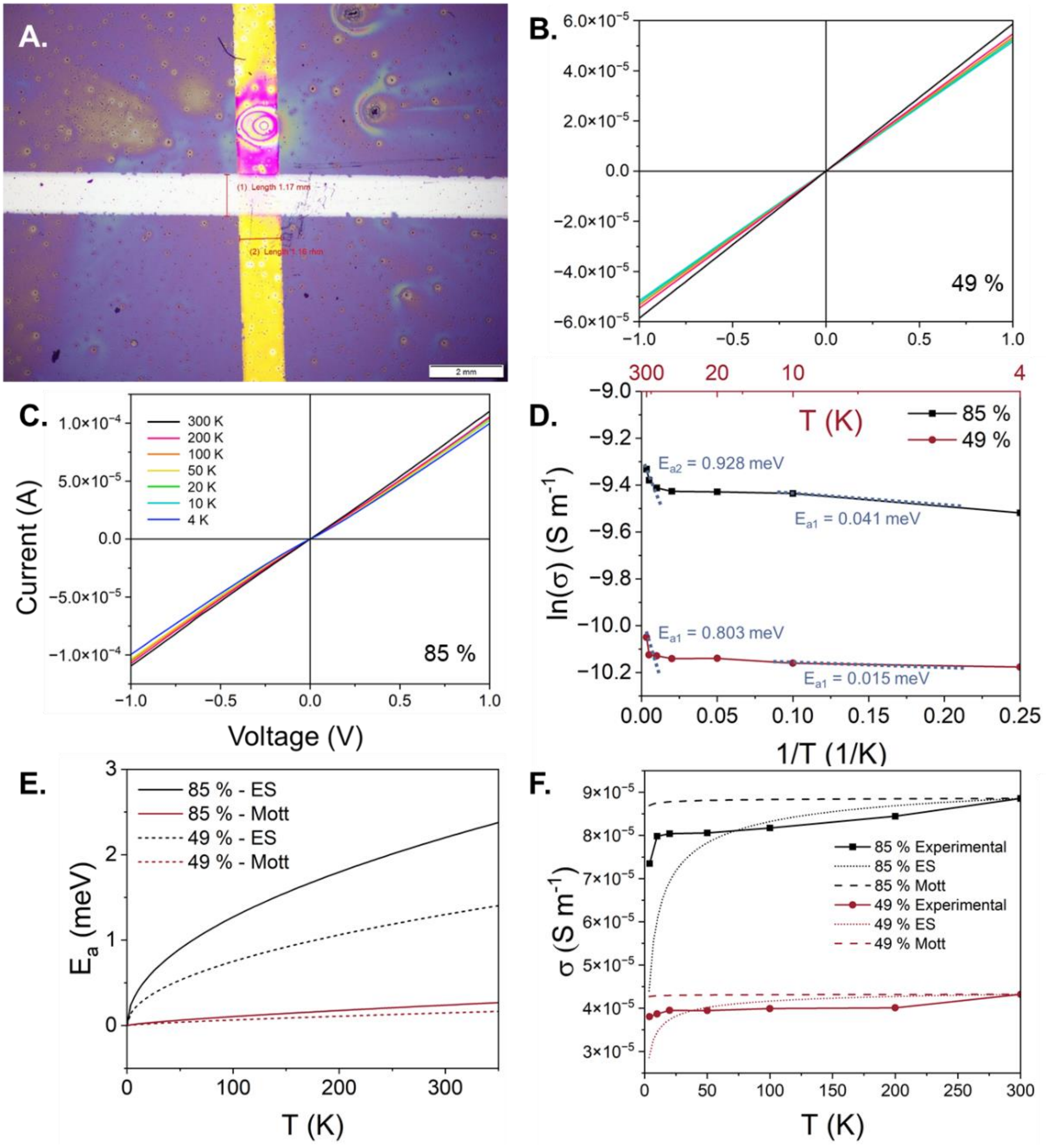

Fig. S17. Charge transport properties of spin valve device made of P1. (A) Top- view image of spin valve took from a polarized optical microscope. Represented I-V curves of (B) 49 % m and (C) 85 % m in various temperatures. (D) ln (σ) vs T^{-1} plot of P1 85 % m (top, black) and 49 % m (bottom, red) at zero field. The linear plots indicate the activation energy calculated at two different temperature regions. (E) Activation energy fitting based on ES and Mott VRH models. (F) σ vs T plots of ES and Mott models based on fittings calculated from (E).

Table S3. Calculated parameters of the VRH models fitted from the experimental results collected from magnetic probe station.

Device	T_0, ES (K)	σ_0, ES (S m^{-1})	T_0, Mott (K)	σ_0, Mott (S m^{-1})
M1 – Single Crystal	31.204	15.665	351.2	12.945
P1 – 85 % *m*	0.103	8.66×10^{-5}	0.0089	9.23×10^{-5}
P2 – 49 % *m*	0.0362	4.13×10^{-5}	0.00131	4.31×10^{-5}

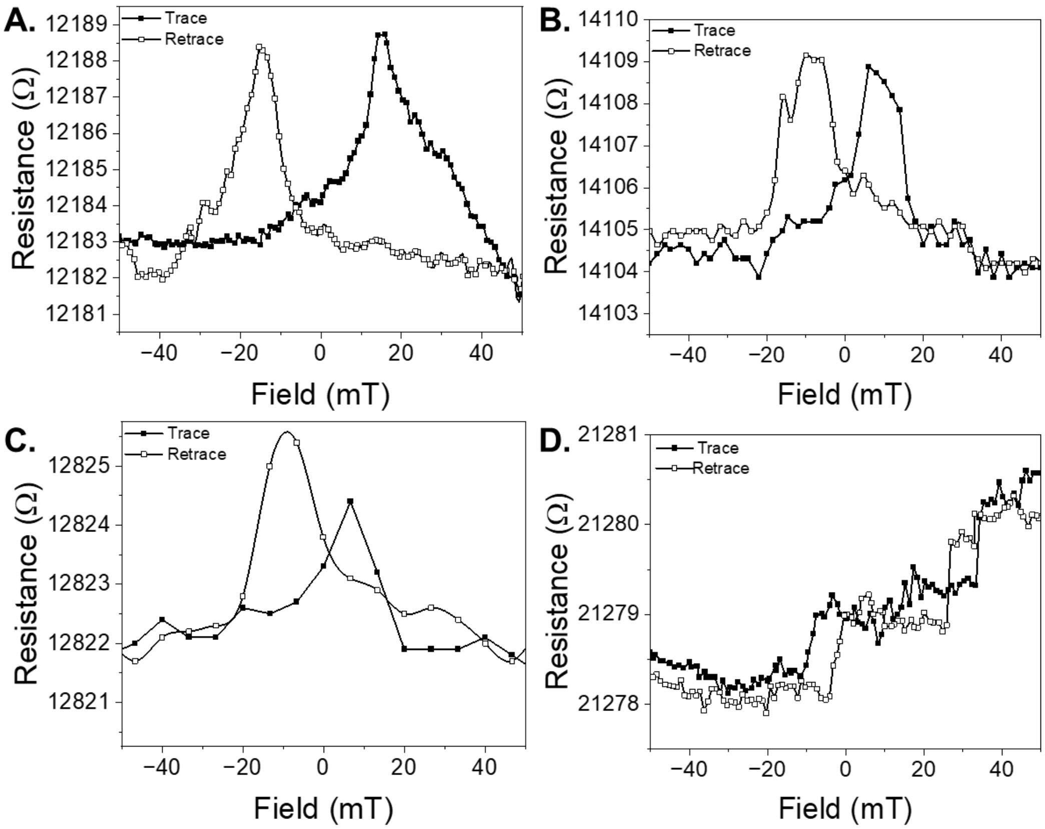

Fig. S18. Raw resistance data of P1 spin valves depending on tacticity. Each datasets represents the trace and retrace values measured from tacticity of (A) 89 %, (B) 78%, (C) 61% and (D) 49 %.

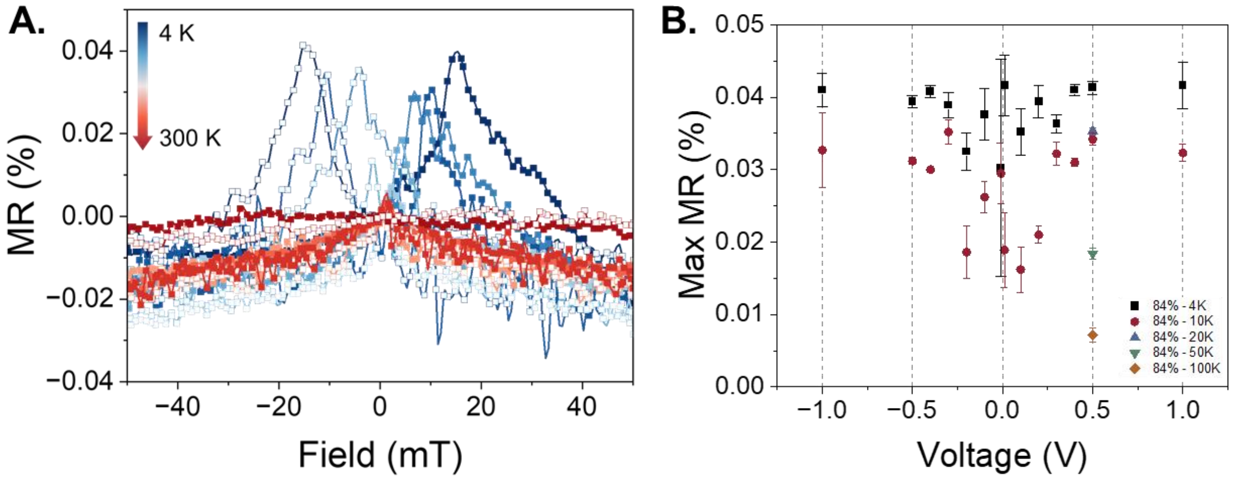

Fig. S19. Additional temperature and voltage dependent MR data. (A) Full MR profile of P1 (85% m) depending on temperature. (B) MR results of 85 % m P1 at various voltage values. P1 does not show voltage dependance behavior. However, depending on the error bars, 0.3 ~ 0.5 V is the most stable voltage region.

Table S4. Resistance and conductivity values of P1-introduced spin valves at room temperature and 4K.

Device	R_{300K} (Ω)	R_{4K} (Ω)	σ_{300K} (S m^{-1})	σ_{4K} (S m^{-1})
49 % *m*	1.35×10^4	2.13×10^4	4.34×10^{-6}	3.48×10^{-6}
61 % *m*	1.02×10^4	1.28×10^4	7.05×10^{-6}	5.78×10^{-6}
78 % *m*	1.05×10^4	1.41×10^4	7.05×10^{-6}	5.25×10^{-6}
85 % *m*	7450	1.22×10^4	9.94×10^{-6}	6.07×10^{-6}

2.5 Powder X-ray Diffraction

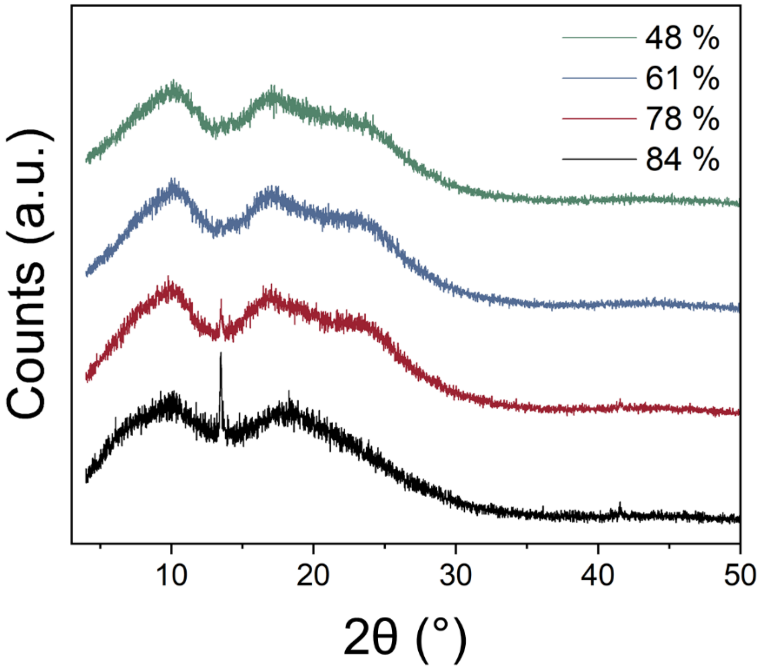

Fig. S20. XRD spectrum of P1. 85 % and 78 % m displays a sharp peak at 13.46 ° while 48 % and 61 % m shows amorphous spectra.

2.6 Computational Methods & Results on Metadynamics Conformational Searching:

Three trimers were analyzed using the Conformer-Rotamer Ensemble Sampling Tool (CREST) and GFN2-xTB semi-empirical potential, both developed by the Grimme group *(44, 45)*. The trimers corresponded to the heterotactic (m,r), syndiotactic (r,r), and isotactic (m,m) triads that map to the atactic, syndiotactic, and atactic polymerization scenarios. The universal force field (UFF) in the OpenBabel quantum chemistry file conversion package was used to generate an initial optimized geometry from stereochemically-specific SMILES strings for each triad *(46),* which was then optimized using xTB (*45*) before being subjected to conformational sampling via CREST using the most comprehensive default setting. The initial geometries and outputted conformers were analyzed to ensure no incorrect bond rearrangements or intramolecular radical dimerization had occurred. Separation (Fig. S23) and alignment (Fig. S22) analysis was performed on all conformations. Each triad has two pairs of nearest neighbors (i.e., the first and second radicals, and the second and third radicals) whose alignment and separation were parsed and reflected in the histograms. Conformer weights within the distributions were calculated via Boltzmann probabilities using the relative energy calculated by CREST for each conformer.

The alignment autocorrelation decay was calculated for each tacticity using the alignment data from the corresponding triads. A virtual polymerization was performed by Boltzmann sampling (i.e., selecting pairs of alignment angles based on the energy of the corresponding triad conformer) the triad statistics 250,000 times, producing a sequence of 500,000 pendant radical alignments. The alignment decay was calculated from these radical sequences using each radical as an independent origin and averaging the alignment dot products with respect to separation along the sequence. This procedure resulted in 499,900 (i.e., 500,000-100) decay curves that were averaged over to yield Fig. 4I The persistence length was reported as the separation after which the alignment fell to e^{-1}. A jupyter notebook illustrating these calculations is distributed with this work.

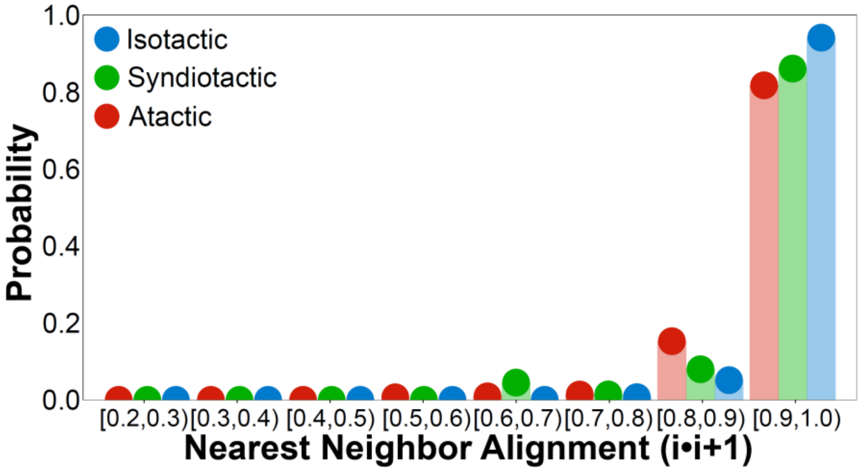

Fig. S21. Alignment analysis of nearest radical neighbor of oxoverdazyl benzyl vinyl ether trimer.

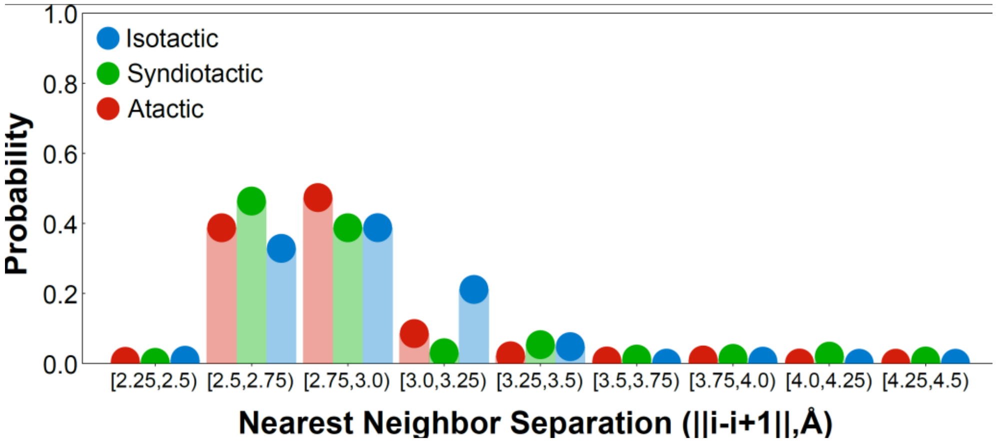

Fig. S22. Separation analysis of nearest radical neighbor of oxoverdazyl benzyl vinyl ether trimer.

2.7 Additional Electrical Conductivity Measurements

Fabricated devices with a geometry of 25 gold channels of a length of 1 mm interdigitated within 1 μm distance were installed to a Lakeshore CRX-VF magnetic probe station. Thin film deposition followed the same conditions of the previous section and thickness profiles were measured. In turn, R values were determined by the same method in the previous section by swiping $- 2 V < V < + 2V$ and measuring the current. To determine the validity of the current responses, a control measurement of the same device with no film, poly(ethylene glycol, PEG) coated, and poly(3,4-ethylenedioxythiophene)-poly(styrenesulfonate, PEDOT:PSS) coated film in room temperature were performed. When reusing the device, sonication of 10 minutes each in chloroform, acetone, isopropyl alcohol was performed, and the devices was submerged in chloroform overnight. Additional examination with a optical microscope was performed to confirm no residual polymers were left on top, then further moved on to the same process. The sequence of studies were done as **P1**(85 % *mm*), **P1**(49 % *mm*), bare, PEG, and PEDOT:PSS. PEDOT:PSS was purchased from Sigma-Aldrich as a conductive grade (1 S m^{-1} conductivity), utilized by adding 20 v% of ethylene glycol and 1 v% of 1-dodecanesulfonic acid as a surfactant for uniform thickness.

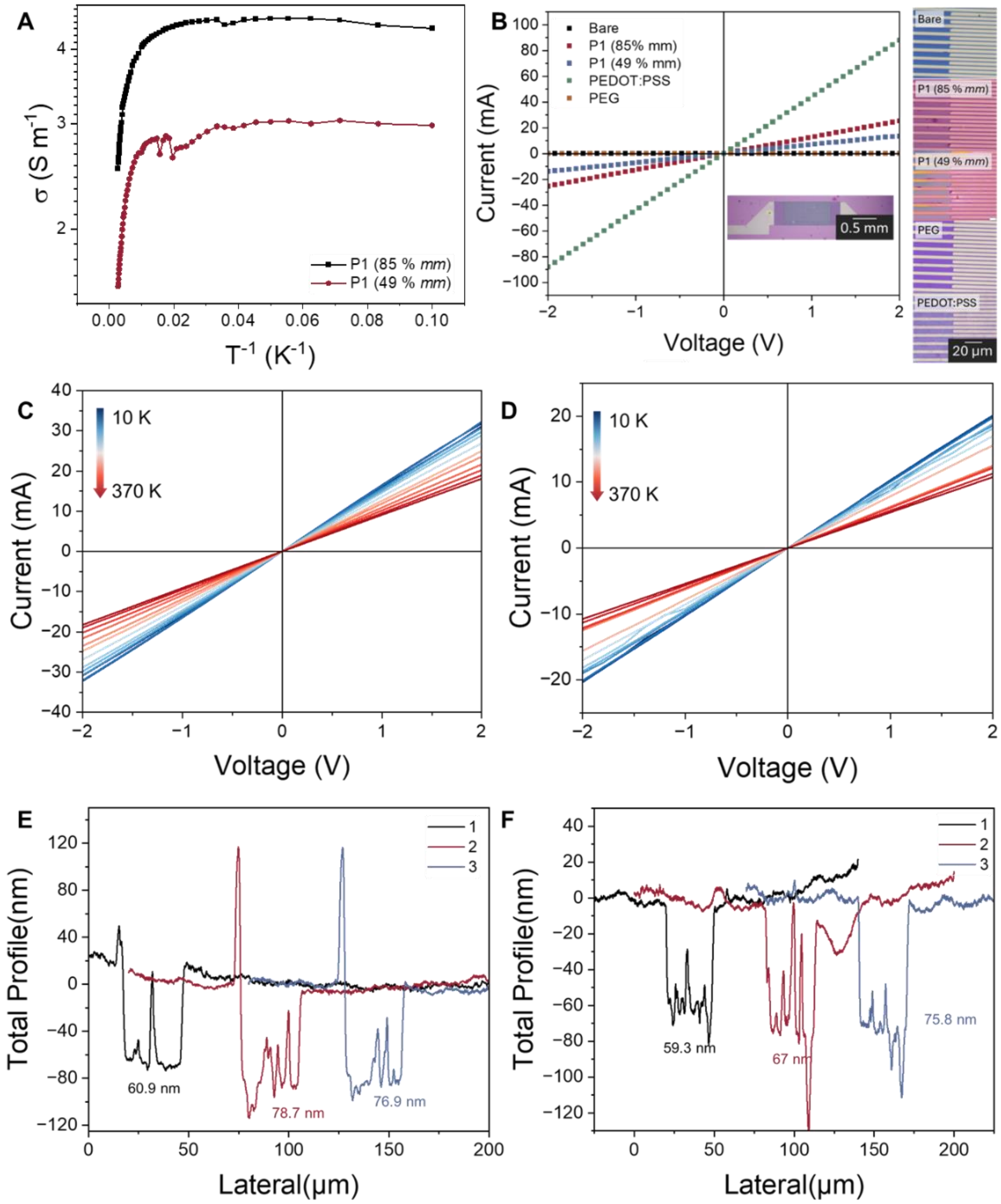

Fig. S23. Charge transport properties of in-plane interdigitated device made of P1. (A) Temperature dependent conductivity profile of P1 (85 % *mm*, black) and P1 (49 % *mm*, red) incorporated to an integrated in-plane device. (B) Comparison of IV profiles measured in room-temperature within a same device. Bare and PEG coated devices displayed only noise signals. IV plots in various temperature were measured when (C) P1 (85 % *mm*) and (D) P1 (49 % *mm*) were coated on top of the device. Thickness profile of (E) P1 (85 % *mm*) (F) P1 (49 % *mm*) were measured three times each, and the average number of these three measurements were used for further calculations.

2.8 Spin Pumping Device Fabrication and Characterization

8.3 mm × 8.3 mm Si/SiO_2 substrates were cleaned in acetone, chloroform, and isopropyl alcohol by sonication for 10 min each in a sequential order. The substrates were then placed in a thermal evaporator installed in inert atmosphere glovebox. 40 nm of NiFe was thermally evaporated on those wafers at a reduced pressure of 10^{-6} bar and rate of 1.0 Å s^{-1} using a shadow mask. After thermal evaporation, substrates were moved to the other side of the glovebox for spin-coating without breaking the vacuum. NiFe is prone to oxidation by air, so all the deposition was carried out in an inert atmosphere glovebox. **P1** was dissolved in chloroform at a concentration of 20 mg ml^{-1}, and was spin-coated on NiFe coated wafers. Films of different thickness of P1 were made by adjusting the spin-coating speed, and the thickness was measured by the Bruker Dektak XT stylus profilometer. The films were then annealed at 50 °C for 10 min to remove the residual solvent. Wafers are then transferred back to the thermal evaporator using the same shadow mask as originally used for NiFe deposition, and 15 nm of Pd is thermally evaporated at a reduced evaporation rate of 0.3 Å s^{-1} to avoid damaging the soft organic **P1** film and avoiding pin hole formation. In another experiment, Pd was replaced by Ag where 15 nm of Ag was thermally evaporated in a similar manner. The wafers are then manually diced into 4 mm × 5 mm size. The FMR response is then measured with the broadband coplanar waveguide (CPW) (NanOSC) using Quantum Design PPMS system and CryoFMR. For FMR measurements, the microwave frequency was varied from 2 GHz – 18 GHz, and corresponding absorption spectra was measured using the lock-in detection technique. Parameters such as effective magnetization and Gilbert damping constant are then extracted from these FMR measurements. To measure ISHE, soldered connections were made on CPW and sample was mounted on those connections using cellophane tape such that Pd comes in great contact with soldered connections. The ISHE voltage is then detected using built in lock-in amplifier, where the measurements were performed by keeping a fixed frequency while sweeping the external magnetic field. As magnetic field (H) is swept through the resonant condition, the maxima of the voltage peak are observed at the same resonant field extracted from FMR measurements. To verify the spin pumping effect, the direction of magnetic field was changed (from + H to – H), and the corresponding signal was measured. In ISHE, magnetic field with opposite direction is expected to produce voltage signal with reversed polarity. A clear change in the sign of voltage is observed for all samples, indicating the presence of spin pumping effect. In these experiments, Pd was used due to its large spin Hall angle which enables the detection of ISHE voltages. Pd was replaced by the low spin Hall angle metal, Ag, where ISHE signal magnitude reduces by 95% indicating that spin detecting contact with large spin Hall angle is essential to fully capture the spin current in our devices.

The dynamic magnetic properties of NiFe/**P1** devices were investigated in a typical ferromagnetic resonance (FMR) experiment, and FMR spectra for bare NiFe and NiFe/**P1** samples were obtained. The direction of the magnetization was fixed by application of external DC magnetic field (H_{dc}) while a transverse radio frequency magnetic field (H_{rf}) drove the magnetization to a non-equilibrium state precessing around magnetic field (H). The dynamics of the precessing magnetization are described by Landau-Lifshitz equation.

$$\frac{\partial M(r,t)}{\partial t} = \gamma \mathrm{M}(\mathrm{r},\mathrm{t}) \times H_{eff}(r,t) + \frac{\alpha_{eff}}{M_s} M(r,t) \times \frac{\partial M(r,t)}{\partial t} \qquad \textbf{(Equation S9)}$$

Here $M(r,t)$ is the magnetization vector; $H_{eff}(r,t)$ is the effective magnetic field, which accounts for external magnetic field, anisotropy field, and exchange field; and M_s is the saturated magnetization. Other important factors are the gyromagnetic ratio (γ) and the Gilbert damping parameter (α). The Gilbert damping parameter is a dimensionless, material-dependent phenomenological parameter that relates the relaxation mechanisms related to magnetization in the Ferromagnetic (FM) system. The first term of **Equation S9** is the conservative term, while the second term is the dissipative term. Thus, with this modified measurement scheme, the derivative of the transmitted power dP/dH_{dc} was measured. This absorption peak can be accurately determined by the following equation.

$$\frac{dP}{dH_{dc}} = K_S \frac{4\Delta H(H-H_{res})}{((\Delta H)^2+4(H-H_{res})^2)^2} + K_{AS} \frac{(\Delta H)^2+4(H-H_{res})}{((\Delta H)^2+4\,(H-H_{res})^2)^2} \qquad \textbf{(Equation S10)}$$

Here, K_s and K_{AS} are the symmetric and anti-symmetric components, respectively. The experimental FMR data were fit to **Equation S10** to determine the resonance field H_{res} and the full width at half maximum (ΔH) of the absorption peak. The frequency-dependent measurements and these two fitting parameters were used to extract $\gamma,\ M_{eff}$, α and the inhomogeneous broadening (ΔH_0). Here, ΔH and f_{res} are related as follows.

$$\Delta H = \frac{4\pi}{\gamma}\alpha_{eff} f_{res} + \Delta H_0 \qquad \textbf{(Equation S11)}$$

Moreover, the resonant frequency (f_{res}) and resonance field (H_{res}) are related by the Kittel equation.

$$f_{res} = \frac{\gamma}{2\pi}\sqrt{H_{res}(H_{res} + 4\pi M_S)} \qquad \textbf{(Equation S12)}$$

Here, the two parameters that are extracted the inhomogeneous damping (ΔH_0) and the Gilbert damping (α). The α parameter is of utmost importance because it describes the magnetization dynamics of the material. This parameter has an intrinsic and extrinsic contribution where the intrinsic contribution accounts for electronic structure of the material and its magnetic interactions (stiffness, exchange, and dipole-dipole interactions). The extrinsic contribution is due to grain boundaries, structural and magnetic inhomogeneity, lattice imperfections and two-magnon scattering. Additionally, spin pumping implies another channel for magnetic relaxation that leads to the enhancement in measured gilbert damping parameter. Generally, ΔH is commonly used to measure the efficiency of spin injection across FM/ non-magnetic (NM) interfaces. Thus, we see in the main text that NiFe/**P1**/Pd have larger α parameter than NiFe alone, implying the leakage of angular momentum during magnetization precession causes this enhancement. For metallic FM/NM multilayers, the magnitude of the pumped spin current can be written as the following.

$$I_S^{pump} = \frac{\hbar}{4\pi}\, g^{\uparrow\downarrow}[\, M \times \frac{\partial M}{\partial t}] \qquad \textbf{(Equation S13)}$$

Here, $g^{\uparrow\downarrow}$ the spin-mixing conduction that depends on the reflection and transmission coefficients of spin current at the FM-NM interface. The pumped spin current, hence, flows perpendicularly to the direction of the applied dc magnetic field. Note that I_S^{pump} in **Equation S13** is proportional to the second term of **Equation S9** according to the theory. The DC component of spin current density, J_s, with ω as the angular frequency of magnetization precession can thus be written as

$$J_s = \frac{\omega}{4\pi}\int_0^{2\pi/\omega} \frac{g^{\uparrow\downarrow}\hbar}{4\pi M_S^2}\left[M \times \frac{\partial M}{\partial t}\right] dt \qquad \textbf{(Equation S14)}$$

Thus, when the NM acts as a perfect spin sink layer, the spin current that flows across FM/NM system is totally expressed by spin mixing conductance. In addition, if the thickness of NM layer is close to its spin diffusion length, then spin accumulation takes place in the NM layer, which could result in the back-flow of spin current into the FM layer. This could significantly affect the resultant ISHE voltages. The back-flow factor can be written as the following.

$$\beta = \frac{\frac{\tau_{Sf}\delta_{sd}}{\hbar}}{\tanh\left(\frac{t_{NM}}{\lambda_{SD}}\right)} \qquad \textbf{(Equation S15)}$$

Here, λ_{SD} denotes spin diffusion length of NM layer, δ_{sd} indicates effective spin-flip scattering energy, and t_{NM} is the thickness of NM layer. Thus, the effective spin mixing conductance takes back-flow factor into account and represents the actual spin injection efficiency across FM/NM layer as follows

$$g_{eff}^{\uparrow\downarrow} = g^{\uparrow\downarrow}\left(\frac{1}{1+\beta g^{\uparrow\downarrow}}\right) \qquad \textbf{(Equation S16)}$$

The spin current then decays in the NM layer as a result of spin diffusion and spin-flip scattering, and the effective spin mixing conductance is reduced due to the back-flow factor. However, recently it was shown that this back-flow factor can be significantly used if trilayer geometry is utilized as compared to bilayer. The spin current density further from the FM/NM interface can then be written as follows.

$$J_s = J_{s0}\sinh[(t_{NM} - y)/\lambda_{SD}]/\sinh[t_{NM}/\lambda_{SD}] \qquad \textbf{(Equation S17)}$$

In this equation, J_{s0} is the spin current density at the FM/NM interface and y is the perpendicular distance from FM/NM interface. In the NM layer, the spin current gets converted to the charge current which develops an electric field that can be detected by the external voltmeter. This charge current can be expressed as the following.

$$J_C = \frac{\Theta_{SH}2e}{\hbar} J_S \times \sigma \qquad \textbf{(Equation S18)}$$

Here, Θ_{SH} is the spin Hall angle (ratio of charge to spin currents), σ is the spin polarization unit vector pointing along the direction of electron's spin and J_S is the spin current density. This charge current then generates the voltage across the sample, that in analogy with FMR, is written as **Equation S19**.

$$V_{meas} = V_S \frac{\Delta H^2}{\Delta H^2+4\,(H-H_{res})^2} + V_{As} \frac{\Delta H(H-H_{res})}{\Delta H^2+4\,(H-H_{res})^2} \qquad \textbf{(Equation S19)}$$

In **Equation S19**, V_s is the symmetric part of the voltage which corresponds to voltage coming from ISHE, and V_{As} is the anti-symmetric part that relate voltage arising from spin rectification effects such as anisotropic magnetoresistance or anomalous Hall effect. The experimental data were fitted to **Equation S19** to investigate the pure spin current transport and any artifacts in the system under study. Now, the FMR linewidth ΔH from FMR data can be used to estimate the effective spin mixing conductance across the FM/NM interface. From FMR measurements, and using **Equation S11**, the change in damping parameter can be written as the following.

$$\Delta\alpha = \alpha_{eff} - \alpha_0 \qquad \textbf{(Equation S20)}$$

Here, α_{eff} is the damping parameter for trilayer NiFe/**P1**/Pd devices while α_0 is the damping parameter for pure NiFe sample. This change can depict how much momentum is lost at the interface. The change in damping parameter can thus be related to effective spin mixing conductance as follows

$$\Delta\alpha = \; g_{eff}^{\uparrow\downarrow}\left(\frac{\gamma\hbar}{4\pi M_s t_{FM}}\right) \qquad \textbf{(Equation S21)}$$

Here $\hbar$ is the Plank's constant and t_{FM} is the thickness of the FM layer. From **Equation S21**, $g_{eff}^{\uparrow\downarrow}$ can be estimated. This $g_{eff}^{\uparrow\downarrow}$ can be further used to estimate spin diffusion length for the material as shown in **Equation S22** below:

$$g_{eff}^{\uparrow\downarrow} = g^{\uparrow\downarrow} \frac{1}{1+\left[\frac{2\sqrt{\epsilon}}{3}\tanh\left(\frac{d_S}{\lambda_{SD}}\right)\right]^{-1}} \qquad \textbf{(Equation S22)}$$

Here d_s is the thickness of non-magnetic spin sink and ϵ is a dimensionless parameter describing material's spin-orbit-coupling energy. While this expression is very useful to utilize in metallic systems with FM/NM structure, the meaning of ϵ in organic materials is not very clear. Thus, we utilized spin decay model to estimate the spin diffusion length of **P1**. Moreover, in trilayer devices such as NiFe/**P1**/Pd, there are two interfaces involved where spin transmission efficiency could get affected. Thus, if one is to utilize **Equation S22** to estimate spin diffusion length, careful determination of spin orbit coupling is required. Similarly, expressions for ISHE

voltage in FM/NM layers have to be modified for trilayer device structures. Based on the spin pumping theory and spin diffusion equation, the model for ISHE in FM/organic material/NM has been formulated. Thus, the charge current generated at the spin-sink NM layer can be written as:

$$I_c = \frac{2eW_F}{\hbar} J_s^{pump} \frac{\Theta_{SHE}^{NM} \lambda_{NM} g_s^{NM} \tanh\left(\frac{d^{NM}}{2\lambda_{NM}}\right) \tanh\left(\frac{d^{NM}}{\lambda_{NM}}\right)}{[g_s^{NM} \tanh\left(\frac{d^{NM}}{\lambda_{NM}}\right) + \frac{g^{\uparrow\downarrow}}{2\pi}] \cosh\left(\frac{d^S}{\lambda_S}\right) + [g_s^S + \frac{g^{\uparrow\downarrow}}{2\pi} \frac{g_s^{NM}}{g_s^S} \tanh\left(\frac{d^{NM}}{\lambda_{NM}}\right) \sinh\left(\frac{d^S}{\lambda^S}\right)} \quad \textbf{(Equation S23)}$$

Where e, $\hbar$ and W_F are elementary charge, reduced Plank's constant, and the width of the ferromagnetic layer. J_s^{pump} is the spin current density, and d^S, λ^S and g_s^S are the thickness, the spin diffusion length and the spin conductivity for organic layer. Similarly, d^{NM} , λ_{NM}, and g_s^{NM} are the thickness, spin diffusion length and spin conductivity for the non-magnetic spin sink layer. By assuming the spin-conductivity of spin-sink layers to be significantly larger than the interface mixing conductivity of FM/organic interface and spin conductivity of **P1**, this can be simplified as the following.

$$I_c = \frac{2eW}{\hbar} J_s^{pump} \frac{\Theta_{\mathrm{SHE}}^{NM} \lambda^{NM} \tanh\left(\frac{d^{NM}}{2\lambda_{NM}}\right)}{\cosh\left(\frac{d^S}{\lambda^S}\right) + \frac{1}{2\pi} \frac{g^{\uparrow\downarrow}}{g_s^S} \sinh\left(\frac{d^S}{\lambda^S}\right)} \quad \textbf{(Equation S24)}$$

Now, as the J_s^{pump}, g_s^S , and $g^{\uparrow\downarrow}$ are not dependent on the spin-sink layer, the total charge current generated due to ISHE in the spin-sink layer scales with the spin diffusion length, spin hall angle and $\tanh\left(\frac{d^{NM}}{2\lambda_{NM}}\right)$ of the spin-sink layer, which is palladium in our case. Thus, the final expression for inverse spin hall voltage for trilayer devices can be written as

$$V_{ISHE} = \frac{2ew}{\hbar} j_s' \frac{\Theta_{\mathrm{SHE}}^{NM} \lambda_{NM} \tanh\left(\frac{d^{NM}}{2\lambda_{NM}}\right)}{d^{NM} \sigma^{NM}} \quad \textbf{(Equation S25)}$$

And here j_s' is a factor term from **Equation S24**. We can define j_s' as follows here

$$j_s' = \frac{J_s^{pump}}{\cosh\left(\frac{d^S}{\lambda^S}\right) + \frac{1}{2\pi} \frac{g^{\uparrow\downarrow}}{g_s^S} \sinh\left(\frac{d^S}{\lambda^S}\right)} \quad \textbf{(Equation S26)}$$

Now for the trilayer devices, when the thickness of organic layer is large and spin lifetime is also large as compared with ferromagnetic layer, the mixing conductance $g^{\uparrow\downarrow}$ at the interface is limited by the spin conductivity and thus $\frac{g^{\uparrow\downarrow}}{g_s^S} \sim 1$. Thus **Equation S26** can then be simplified to

$$j_s' = \frac{J_s^{pump}}{\cosh\left(\frac{d^S}{\lambda^S}\right) + \sinh\left(\frac{d^S}{\lambda^S}\right)} = J_s^{pump} \exp\left(-\frac{d^S}{\lambda^S}\right) \quad \textbf{(Equation S27)}$$

Thus, we see that the spin current in the organic layer follows the exponential decay. In turn, the following holds.

$$V_{ISHE} \propto \exp\left(-\frac{d^S}{\lambda^S}\right) \quad \textbf{(Equation S28)}$$

This adjustment leads to reliable fit with reduced errors, indicating improved accuracy in the results following the fit as below.

$$V_{ISHE} = A\, V_{ISHE,max} \exp\left(-\frac{d^S}{\lambda^S}\right) \quad \textbf{(Equation S29)}$$

Here, A is an arbitrary constant. This also correlates well with the thickness dependent ISHE data where the voltage seems to drop as the thickness of **P1** increases. Thus, we can assume that spin diffusion length, λ_s of **P1** can be determined using **Equation S28**.

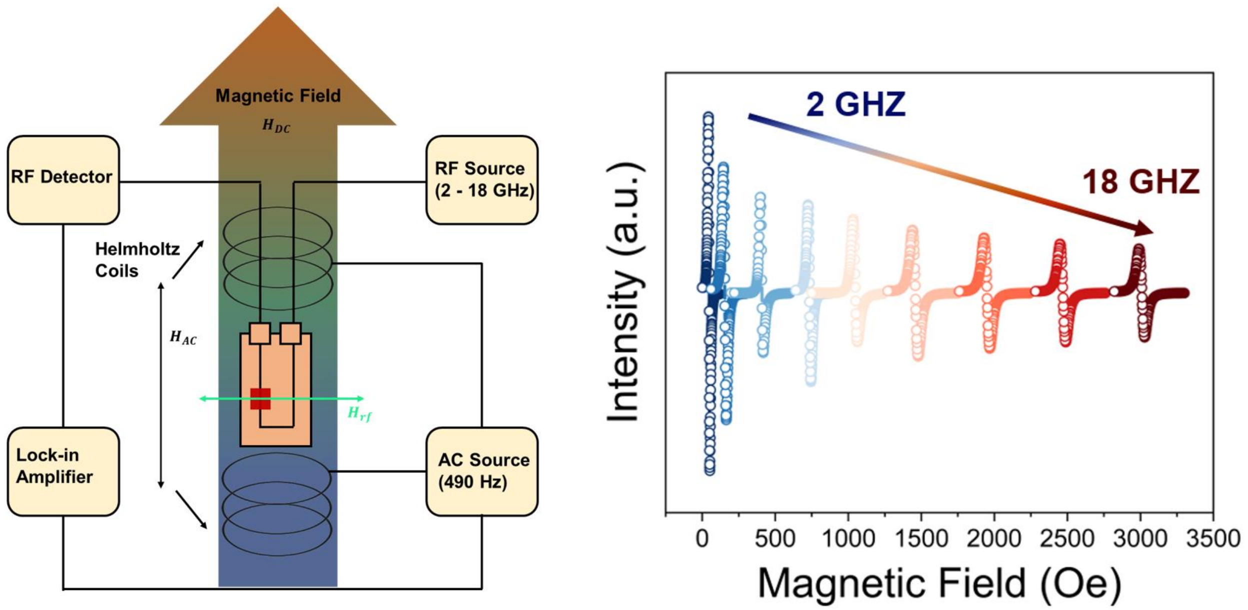

Fig. S24. Additional description of spin pump experimental methods and data. (A) Experimental schematics for FMR measurement with radio frequency (RF) source, RF detector for radio frequency detection, and an AC source for field modulation. The big arrow shows the external H_{dc} in-plane field. The sample (red square) is placed on a coplanar waveguide, and a small radio field is applied to generate the precessing motion. (B) FMR spectrum (2 GHz – 18 GHz) for NiFe/P1/Pd bilayer system. The presence of P1 causes broadening in FMR spectrum due to dissipation of spin current from NiFe into P1.

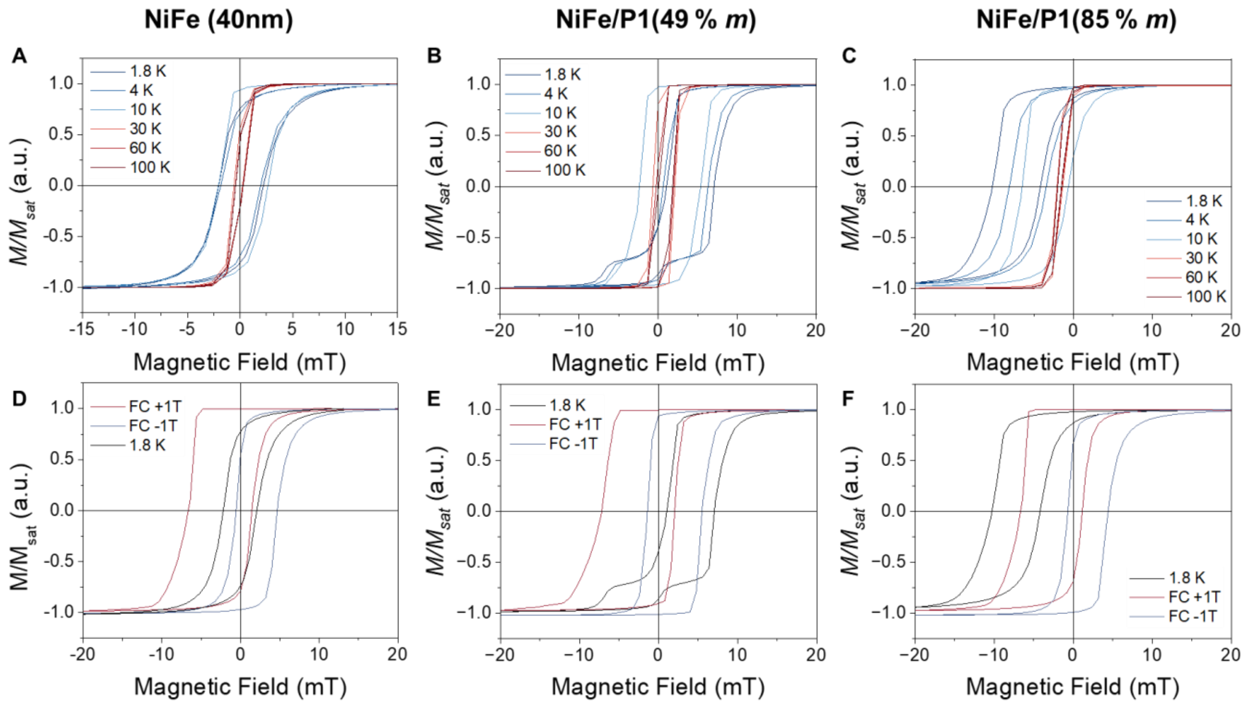

Fig. S26. *M* vs *H* plot of thin films to study the interfacial effect. M/M_{sat} profiles of (A) NiFe (40 nm), (B) NiFe/P1 (49 % *m*), and (C) NiFe/P1 (85 % *m*). Additional observation of field-cooled measurements was performed in +1 T (red) and – 1 T (blue) in the same sequential order (D, E, and F). Zero-field cooling M/M_{sat} profiles of NiFe thin films (A, D) were centered to the origin, while as the other remained as raw data including experimental artifacts.

2.9 X-ray crystallography information

6-oxo-1,5-diphenyl-3-(4-((vinyloxy)methyl)phenyl)-5,6-dihydro-1H-1,2,4,5-tetrazin-2-yl

CCDC#: 2324183

6-oxo-1,5-diphenyl-3-(4-((vinyloxy)methyl)phenyl)-5,6-dihydro-1H-1,2,4,5-tetrazin-2-yl were crystallized via convection crystallization from methanol (Fig. S24). Various attempts at crystallization were made, but ultimately the only suitable crystals were thin and flexible. A suitable crystal with dimensions of 0.010 x 0.020 x 0.070 mm was mounted from Paratone on a MiTeGin loop. Data was collected using phi and omega scans at 150.0 K with a *Bruker D8 VENTURE* diffractometer using Cu Ka at a resolution of 0.84 Å. Data reduction was performed using *SAINT* (SAINT, Bruker Analytical X-Ray Systems, Madison, WI.). Data were corrected for absorption using the multi-scan method (SADABS). The structure was solved using Superflip and the model was refined using Oxford CRYSTALS. All non-hydrogen atoms were refined anisotropically. Hydrogen atoms were generated on the ideal positions and refined as riding atoms.

Disorder was observed on the position of the vinyl ether but was not modeled due to the low data to parameter ratio.

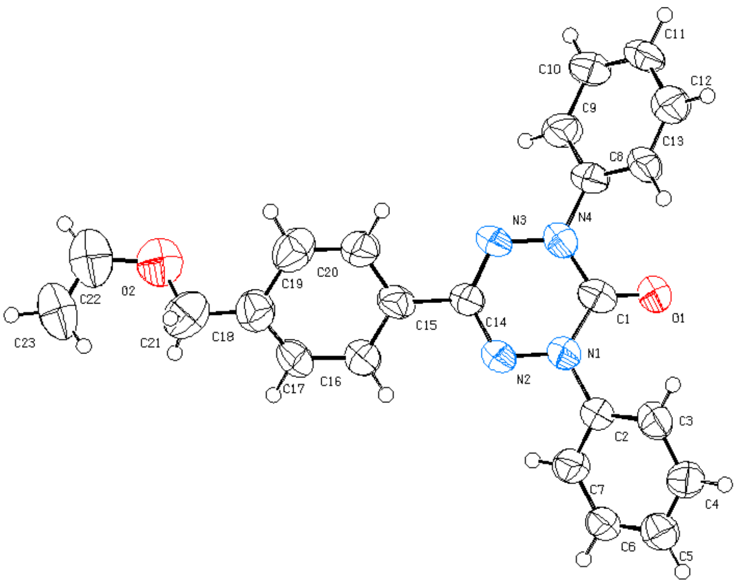

Fig. S25. Structure and solved crystal structure of 6-oxo-1,5-diphenyl-3-(4-((vinyloxy)methyl)phenyl)-5,6-dihydro-1H-1,2,4,5-tetrazin-2-yl (M1).

Experimental. Purple needles of M1

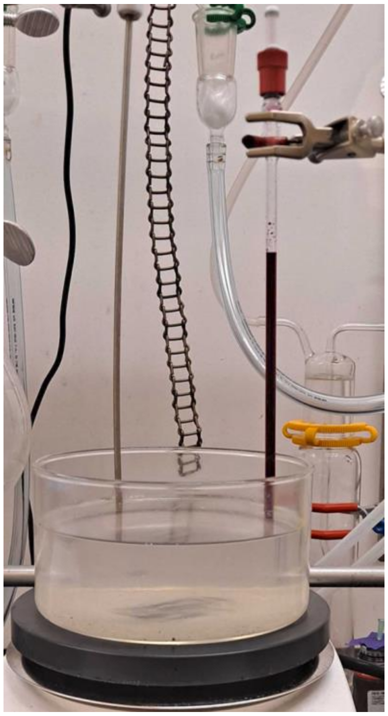

Fig. S26. Convection crystallization of M1 in methanol in a J-young tube.

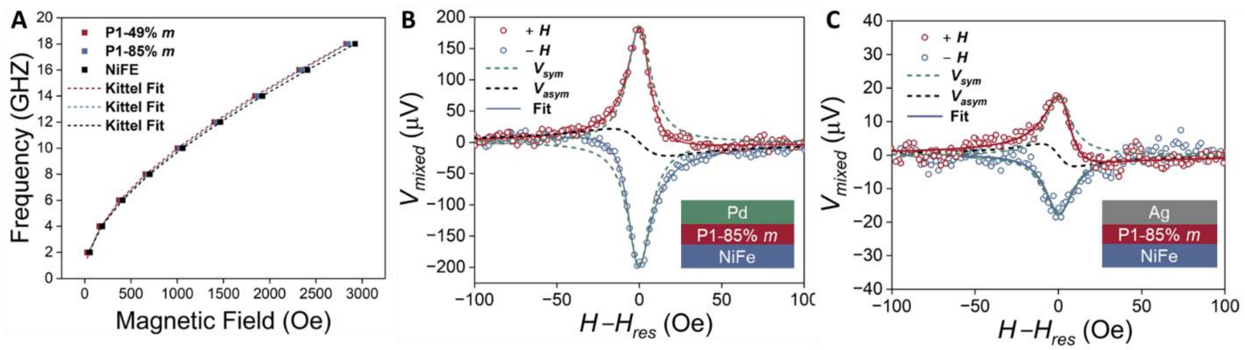

Fig. S29. Additional spin device information for understanding spin transport. (A) Frequency vs. field data **P1-**49% m, **P1-**85% m, and pure NiFe electrode fitted to Kittel Eq. S12. **(B)** ISHE response for **P1-**85% m at 4 GHz with Pd as spin sink layer, where ISHE voltage is flipped for **P1-**85% m compared to **P1**-49% m for a field applied in same direction. **(C)** ISHE response for **P1-**85% m at 4 GHz with Ag as a spin sink layer.

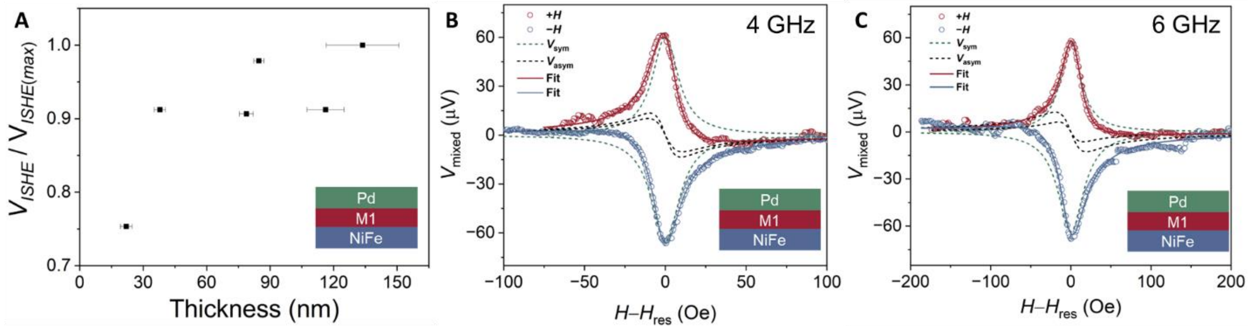

Fig. S30. ISHE measurements of M1. (A) M1 does not show a trend of V_{ISHE} vs. thickness, indicating that they do not act as long spin transporting material. However, ISHE plots are achievable in **(B)** 4 GHz and **(C)** 6 GHz showing that ISHE voltage can be achieved due to conducting nature of **M1**.

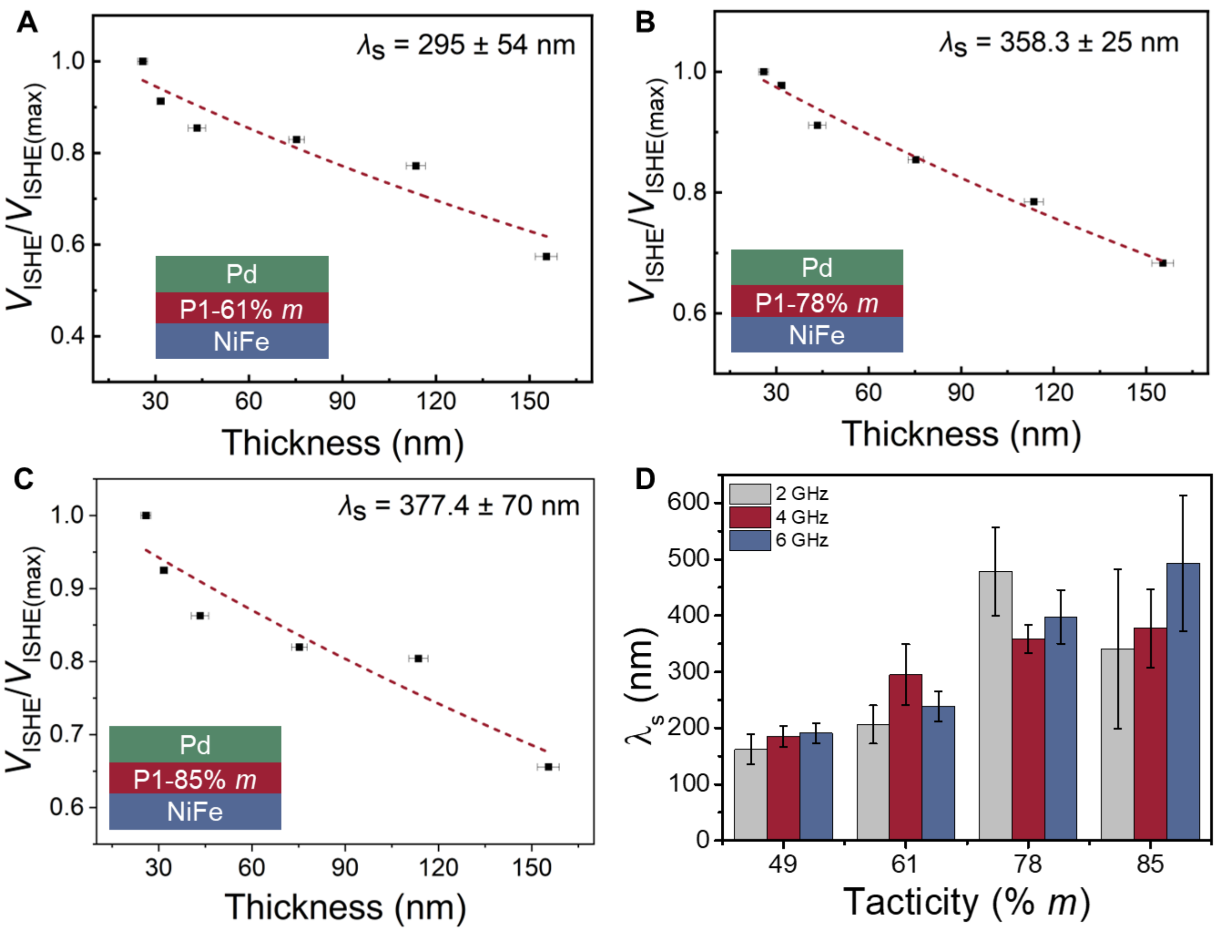

Fig S31. Spin diffusion length calculations of P1. V_{ISHE} vs. thickness for **(A) P1**-61% *m*, **(B) P1**-78% *m*, and **(C) P1**-85% *m* fitted to exponential decay function to extract spin diffusion length. **(D)** This trend of longer λs to higher stereoregularity was consistent in other frequencies, representing the reproducibility.

Crystal Data. $C_{23}H_{20}N_4O_2$, M_w = 384.43 g.mol^{-1}, monoclinic, *P2₁/C*, a = 21.6519(13) Å, b = 3.8276(3) Å, c = 22.3894(13) Å, a = 90.000^{o}, b = 96.015(5)^{o}, g = 90.000^{o}, *V* = 1845.32(20) Å^3, *T* = 150.0 K, *Z* = 4, *Z'* = 1, 22015 reflections measured, 11180 unique, 2766 above 20 s(I) which were used in all calculations. For structure refinement there were 262 parameters and 15 restraints with a final GooF = 0.996, R1 = 0.1661, and wR2 = 0.4324.

Atom Coordinates and Uiso or Ueq:

TABLE S5. Atomic coordinates (x 10^4) and equivalent isotropic displacement parameters for 23143. U(eq) is defined as one third of the trace of the orthogonalized Uij tensor.

**Atom**	**x**	**y**	**x**	**U(eq)** (Å^{2}E3)
N(1)	1599(4)	520(30)	2113(4)	58
N(2)	2083(4)	1800(30)	1861(3)	61
N(3)	2621(4)	2030(30)	2869(3)	60
N(4)	2119(4)	670(30)	3105(4)	73
C(1)	1605(5)	-380(30)	2731(5)	58
C(2)	1073(4)	-40(30)	1692(4)	60
C(3)	511(5)	1030(40)	1860(5)	70
C(4)	15(5)	420(30)	1441(5)	65
C(5)	70(5)	-1120(30)	866(5)	68
C(6)	621(5)	-1990(30)	713(5)	61
C(7)	1128(5)	-1550(30)	1112(5)	63
C(8)	2159(5)	270(40)	3763(4)	65
C(9)	2675(5)	-1420(40)	4057(5)	75
C(10)	2728(5)	-1820(30)	4701(5)	64
C(11)	2272(5)	-600(40)	5033(5)	82
C(12)	1766(5)	1020(40)	4720(5)	75
C(13)	1708(5)	1400(40)	4097(5)	68
C(14)	2576(4)	2390(30)	2256(4)	49
C(15)	3126(5)	3340(30)	1986(4)	60
C(16)	3193(5)	2690(30)	1356(5)	55
C(17)	3719(5)	3740(40)	1115(5)	68
C(18)	4184(3)	5640(40)	1415(5)	78
C(19)	4112(5)	5980(40)	2101(7)	82
C(20)	3596(5)	4970(40)	2345(5)	70
C(21)	4711(3)	6580(40)	1134(5)	93
C(22)	5763(4)	6620(40)	1285(6)	114

C(23)	5766(6)	7780(40)	705(6)	101
		-		
O(1)	1199(3)	2050(30)	2938(3)	72
O(2)	5228(3)	5090(30)	1482(4)	112

TABLE S6. Bond lengths for 23143.

**Bond**	**Length** (Å)
N(1)-N(2)	1.355(11)
N(1)-C(1)	1.381(13)
N(1)-C(2)	1.426(13)
N(2)-C(14)	1.343(12)
N(3)-N(4)	1.385(12)
N(3)-C(14)	1.327(11)
N(4)-C(1)	1.394(14)
N(4)-C(8)	1.427(13)
C(1)-O(1)	1.233(13)
C(2)-C(3)	1.407(15)
C(2)-C(7)	1.401(15)
C(3)-C(4)	1.379(16)
C(3)-H(31)	0.95
C(4)-C(5)	1.395(16)
C(4)-H(41)	0.95
C(5)-C(6)	1.351(15)
C(5)-H(51)	0.95
C(6)-C(7)	1.361(14)
C(6)-H(61)	0.95
C(7)-H(71)	0.95
C(8)-C(9)	1.415(15)
C(8)-C(13)	1.373(14)
C(9)-C(10)	1.397(14)
C(9)-H(91)	0.95
C(10)-C(11)	1.389(16)
C(10)-H(101)	0.95
C(11)-C(12)	1.403(17)
C(11)-H(111)	0.95
C(12)-C(13)	1.350(14)
C(12)-H(121)	0.95
C(13)-H(131)	0.95
C(14)-C(15)	1.462(14)
C(15)-C(16)	1.412(13)
C(15)-C(20)	1.389(15)
C(16)-C(17)	1.396(14)
C(16)-H(161)	0.95
C(17)-C(18)	1.375(16)
C(17)-H(171)	0.95
C(18)-C(19)	1.517(17)

C(18)-C(21)	1.4301(5)
C(19)-C(20)	1.374(17)
C(19)-H(191)	0.95
C(20)-H(201)	0.95
C(21)-O(2)	1.4299(5)
C(21)-H(211)	0.95
C(21)-H(212)	0.95
C(22)-C(23)	1.332(5)
C(22)-O(2)	1.440(5)
C(22)-H(221)	0.95
C(23)-H(231)	0.95
C(23)-H(232)	0.95

TABLE S7. Bond angles for 23143.

**Bond**	**Angle** (°)
N(2)-N(1)-C(1)	123.6(8)
N(2)-N(1)-C(2)	116.0(8)
C(1)-N(1)-C(2)	120.3(9)
N(1)-N(2)-C(14)	116.3(8)
N(4)-N(3)-C(14)	115.4(8)
N(3)-N(4)-C(1)	123.1(8)
N(3)-N(4)-C(8)	116.0(9)
C(1)-N(4)-C(8)	120.8(9)
N(4)-C(1)-N(1)	114.5(10)
N(4)-C(1)-O(1)	122.1(9)
N(1)-C(1)-O(1)	123.3(10)
N(1)-C(2)-C(3)	119.5(10)
N(1)-C(2)-C(7)	119.4(9)
C(3)-C(2)-C(7)	121.1(10)
C(2)-C(3)-C(4)	117.5(11)
C(2)-C(3)-H(31)	120.6
C(4)-C(3)-H(31)	121.8
C(3)-C(4)-C(5)	121.2(11)
C(3)-C(4)-H(41)	117.9
C(5)-C(4)-H(41)	120.9
C(4)-C(5)-C(6)	119.3(11)
C(4)-C(5)-H(51)	120.2
C(6)-C(5)-H(51)	120.5
C(5)-C(6)-C(7)	122.5(10)
C(5)-C(6)-H(61)	120.1
C(7)-C(6)-H(61)	117.4
C(2)-C(7)-C(6)	118.3(10)
C(2)-C(7)-H(71)	119.6
C(6)-C(7)-H(71)	122.1
N(4)-C(8)-C(9)	117.5(10)
N(4)-C(8)-C(13)	121.2(10)
C(9)-C(8)-C(13)	121.2(10)
C(8)-C(9)-C(10)	118.6(10)
C(8)-C(9)-H(91)	120.3
C(10)-C(9)-H(91)	121.2
C(9)-C(10)-C(11)	119.4(10)
C(9)-C(10)-H(101)	120.3
C(11)-C(10)-H(101)	120.2
C(10)-C(11)-C(12)	119.9(10)

C(10)-C(11)-H(111)	120.5
C(12)-C(11)-H(111)	119.6
C(11)-C(12)-C(13)	121.3(11)
C(11)-C(12)-H(121)	119.7
C(13)-C(12)-H(121)	119
C(8)-C(13)-C(12)	119.6(11)
C(8)-C(13)-H(131)	118.7
C(12)-C(13)-H(131)	121.7
N(2)-C(14)-N(3)	126.2(9)
N(2)-C(14)-C(15)	117.2(8)
N(3)-C(14)-C(15)	116.5(9)
C(14)-C(15)-C(16)	121.5(9)
C(14)-C(15)-C(20)	120.6(9)
C(16)-C(15)-C(20)	117.9(10)
C(15)-C(16)-C(17)	119.4(10)
C(15)-C(16)-H(161)	119.9
C(17)-C(16)-H(161)	120.8
C(16)-C(17)-C(18)	127.0(9)
C(16)-C(17)-H(171)	117.4
C(18)-C(17)-H(171)	115.6
C(17)-C(18)-C(19)	110.9(9)
C(17)-C(18)-C(21)	123.4(11)
C(19)-C(18)-C(21)	124.9(10)
C(18)-C(19)-C(20)	122.1(11)
C(18)-C(19)-H(191)	118.4
C(20)-C(19)-H(191)	119.5
C(15)-C(20)-C(19)	122.0(11)
C(15)-C(20)-H(201)	119.5
C(19)-C(20)-H(201)	118.5
C(18)-C(21)-O(2)	109.499(11)
C(18)-C(21)-H(211)	110.3
O(2)-C(21)-H(211)	115.1
C(18)-C(21)-H(212)	106.9
O(2)-C(21)-H(212)	105.1
H(211)-C(21)-H(212)	109.5
C(23)-C(22)-O(2)	120.10(10)
C(23)-C(22)-H(221)	113.4
O(2)-C(22)-H(221)	123.6
C(22)-C(23)-H(231)	126.2
C(22)-C(23)-H(232)	113.4

H(231)-C(23)-H(232)	120
C(22)-O(2)-C(21)	109.502(11)

Data S1 and S2.

Magnetic persistence calculations, along with the complete set of raw data on energy, separation, and alignment used to generate all plots in the associated DRYAD repository, are provided in the below link.

https://doi.org/10.5061/dryad.44j0zpcr2

This folder contains both the raw data files (.p files, Data S1) and the calculation code (.ipynb file, Data S2). Supplementary crystallography data of M1, that can also be accessible in CCDC (#: 2324183) is included.

REFERENCES AND NOTES

1. I. Žutić, J. Fabian, S. Das Sarma, Spintronics: Fundamentals and applications. *Rev. Mod. Phys.* **76**, 323–410 (2004).

2. A. V. Chumak, V. I. Vasyuchka, A. A. Serga, B. Hillebrands, Magnon spintronics. *Nat. Phys.* **11**, 453–461 (2015).

3. P. Ghising, C. Biswas, Y. H. Lee, Graphene spin valves for spin logic devices. *Adv. Mater.* **35**, 2209137 (2023).

4. H. Liu, C. Zhang, H. Malissa, M. Groesbeck, M. Kavand, R. McLaughlin, S. Jamali, J. Hao, D. Sun, R. A. Davidson, L. Wojcik, J. S. Miller, C. Boehme, Z. Valy Vardeny, Organic-based magnon spintronics. *Nat. Mater.* **17**, 308–312 (2018).

5. N. Zheng, H. Liu, Y.-J. Zeng, Dynamical behavior of pure spin current in organic materials. *Adv. Sci.* **10**, 2207506 (2023).

6. D. Sun, K. J. Van Schooten, M. Kavand, H. Malissa, C. Zhang, M. Groesbeck, C. Boehme, Z. Valy Vardeny, Inverse spin Hall effect from pulsed spin current in organic semiconductors with tunable spin-orbit coupling. *Nat. Mater.* **15**, 863–869 2016.

7. D. Li, G. Yu, Innovation of materials, devices, and functionalized interfaces in organic spintronics. *Adv. Funct. Mater.* **31**, 2100550 (2021).

8. Y. Joo, V. Agarkar, S. H. Sung, B. M. Savoie, B. W. Boudouris, A nonconjugated radical polymer glass with high electrical conductivity. *Science* **359**, 1391–1395 (2018).

9. Y. Tan, S. N. Hsu, H. Tahir, L. Dou, B. M. Savoie, B. W. Boudouris, Electronic and spintronic open-shell macromolecules, quo vadis? *J. Am. Chem. Soc.* **144**, 626–647 (2022).

10. S. Akkiraju, D. M. Gilley, B. M. Savoie, B. W. Boudouris, Anomalous magnetoresistance in a nonconjugated radical polymer glass. *Proc. Natl. Acad. Sci. U.S.A.* **120**, e2308741120 (2023).

11. Z. Liang, S. N. Hsu, Y. Tan, H. Tahir, H. J. Kim, K. Liu, J. F. Stoehr, M. Zeller, L. Dou, B. M. Savoie, B. W. Boudouris, Significant charge transport effects due to subtle molecular changes in nitroxide radical single crystals. *Cell Rep. Phys. Sci.* **4**, 101409 (2023).

12. W. J. M. Naber, S. Faez, W. G. Van Der Wiel, Organic spintronics. *J. Phys. D Appl. Phys.* **40**, R205 (2007).

13. S. A. Wolf, D. D. Awschalom, R. A. Buhrman, J. M. Daughton, S. Von Molnár, M. L. Roukes, A. Y. Chtchelkanova, D. M. Treger, Spintronics: A spin-based electronics vision for the future. *Science* **294**, 1488–1495 (2001).

14. M. Suguro, S. Iwasa, Y. Kusachi, Y. Morioka, K. Nakahara, Cationic polymerization of poly(vinyl ether) bearing a TEMPO radical: A new cathode-active material for organic radical batteries. *Macromol. Rapid Commun.* **28**, 1929–1933 (2007).

15. Y. Tan, B. W. Boudouris, B. M. Savoie, Bridging the monomer to polymer gap in radical polymer design. *ACS Macro Lett.* **12**, 801–807 (2023).

16. F. A. Neugebauer, H. Fischer, 6-Oxoverdazyls. *Angew. Chem. Int. Ed. Engl.* **19**, 724–725 (1980).

17. F. A. Neugebauer, H. Fischer, C. Krieger, Verdaryls. Part 33. EPR and ENDOR Studies of 6-oxo-and 6-thioxoverdazyls. X-ray molecular structure of 1,3,5-triphenyl-6-oxoverdazyl and 3-tert-butyl-1,5-diphenyl-6-thioxoverdazyl. *J. Chem. Soc. Perkin 1* **2**, 535–544 (1993).

18. M. Kertesz, Pancake bonding: An unusual pi-stacking interaction. *Chem. A Eur. J.* **25**, 400–416 (2019).

19. J. Xie, S. Ewing, J.-N. Boyn, A. S. Filatov, B. Cheng, T. Ma, G. L. Grocke, N. Zhao, R. Itani, X. Sun, H. Cho, Z. Chen, K. W. Chapman, S. N. Patel, D. V. Talapin, J. Park, D. A. Mazziotti, J. S. Anderson, Intrinsic glassy-metallic transport in an amorphous coordination polymer. *Nature* **611**, 479–484 (2022).

20. P. Stallinga, Electronic transport in organic materials: Comparison of band theory with percolation/(variable range) hopping theory. *Adv. Mater.* **23**, 3356–3362 (2011).

21. Z. Liang, Y. Tan, S. N. Hsu, J. F. Stoehr, H. Tahir, A. B. Woeppel, S. Debnath, M. Zeller, L. Dou, B. M. Savoie, B. W. Boudouris, Charge transport and antiferromagnetic ordering in nitroxide radical crystals. *Mol. Syst. Des. Eng.* **8**, 464–472 (2022).

22. S. W. Jiang, D. J. Shu, L. Lin, Y. J. Shi, J. Shi, H. F. Ding, J. Du, M. Wang, D. Wu, Strong asymmetrical bias dependence of magnetoresistance in organic spin valves: The role of ferromagnetic/organic interfaces. *New J. Phys.* **16**, 013028 (2014).

23. J. Danon, X. Wang, A. Manchon, Pauli spin blockade and the ultrasmall magnetic field effect. *Phys. Rev. Lett.* **111**, 66802 (2013).

24. H. Tahir, N. Eedugurala, S.-N. Hsu, P. Mahalingavelar, B. M. Savoie, B. W. Boudouris, J. D. Azoulay, Large room-temperature magnetoresistance in a high-spin donor–acceptor conjugated polymer. *Adv. Mater.* **36**, 2306389 (2024).

25. C. Wang, H. Hao, K. Tajima, Essential role of triplet diradical character for large magnetoresistance in Quinoidal organic semiconductor with high electron mobility. *Adv. Sci.* **9**, 2201045 (2022).

26. N. J. Harmon, M. E. Flatté, Organic magnetoresistance from deep traps. *J. Appl. Phys.* **116**, 043707 (2014).

27. C. C. Sorensen, C. T. Kozuszek, M. A. Borden, F. A. Leibfarth, Asymmetric ion-pairing in stereoselective vinyl polymerization. *ACS Catal.* **13**, 3272–3284 (2023).

28. N. Tsuji, J. L. Kennemur, T. Buyck, S. Lee, S. Prévost, P. S. J. Kaib, D. Bykov, C. Farès, B. List, Activation of olefins via asymmetric Brønsted acid catalysis. *Science* **359**, 1501–1505 (2018).

29. S. K. Nistanaki, C. G. Williams, B. Wigman, J. J. Wong, B. C. Haas, S. Popov, J. Werth, M. S. Sigman, K. N. Houk, H. M. Nelson, Catalytic asymmetric C–H insertion reactions of vinyl carbocations. *Science* **378**, 1085–1091 (2022).

30. P. C. Knutson, A. J. Teator, T. P. Varner, C. T. Kozuszek, P. E. Jacky, F. A. Leibfarth, Brønsted acid catalyzed stereoselective polymerization of vinyl ethers. *J. Am. Chem. Soc.* **143**, 16388–16393 (2021).

31. Z. Yang, X. Zhang, Y. Jiang, Q. Ma, S. Liao, Organocatalytic stereoselective cationic polymerization of vinyl ethers by employing a confined Brønsted acid as the catalyst. *Sci. China Chem.* **65**, 304–308 (2022).

32. X. Zhang, Z. Yang, Y. Jiang, S. Liao, Organocatalytic, stereoselective, cationic reversible addition–fragmentation chain-transfer polymerization of vinyl ethers. *J. Am. Chem. Soc.* **144**, 679–684 (2022).

33. H. Watanabe, T. Yamamoto, A. Kanazawa, S. Aoshima, Stereoselective cationic polymerization of vinyl ethers by easily and finely tunable titanium complexes prepared from tartrate-derived diols: Isospecific polymerization and recognition of chiral side chains. *Polym. Chem.* **11**, 3398–3403 (2020).

34. C. C. Sorensen, F. A. Leibfarth, Stereoselective helix-sense-selective cationic polymerization of *N*-vinylcarbazole using chiral Lewis acid catalysis. *J. Am. Chem. Soc.* **144**, 8487–8492 (2022).

35. A. J. Teator, F. A. Leibfarth, Catalyst-controlled stereoselective cationic polymerization of vinyl ethers. *Science* **363**, 1439–1443 (2019).

36. C. E. Schildknecht, A. O. Zoss, C. McKinley, Vinyl alkyl ethers. *Ind. Eng. Chem.* **39**, 180–186 (1947).

37. S. Mugiraneza, A. M. Hallas, Tutorial: A beginner’s guide to interpreting magnetic susceptibility data with the Curie-Weiss law *Commun. Phys.* **5**, 95 (2022).

38. A. Rajca, Organic diradicals and polyradicals: From spin coupling to magnetism? *Chem. Rev.* **94**, 871–893 (1994).

39. Z. H. Xiong, D. Wu, Z. V. Vardeny, J. Shi, Giant magnetoresistance in organic spin-valves. *Nature* **427**, 821–824 (2004).

40. A. J. Drew, J. Hoppler, L. Schulz, F. L. Pratt, P. Desai, P. Shakya, T. Kreouzis, W. P. Gillin, A. Suter, N. A. Morley, V. K. Malik, A. Dubroka, K. W. Kim, H. Bouyanfif, F. Bourqui, C. Bernhard, R. Scheuermann, G. J. Nieuwenhuys, T. Prokscha, E. Morenzoni, Direct measurement of the electronic spin diffusion length in a fully functional organic spin valve by low-energy muon spin rotation. *Nat. Mater.* **8**, 109–114 (2009).

41. N. Zheng, X. Wang, Y. Zheng, D. Li, Z. Lin, W. Zhang, K.-J. Jin, G. Yu, Negative magnetoresistance behavior in polymer spin valves based on donor−Acceptor conjugated molecules. *Adv. Mater. Interfaces* **7**, 2000868 (2020).

42. E. C. Ahn, 2D materials for spintronic devices. *Npj 2D Mater. Appl.* **4**, 17 (2020).

43. T. Varner, "Stereoselective cationic polymerization of vinyl ethers through asymmetric ion-pairing catalysis," thesis, University of North Carolina at Chapel Hill, NC (2021).

44. P. Pracht, F. Bohle, S. Grimme, Automated exploration of the low-energy chemical space with fast quantum chemical methods. *Phys. Chem. Chem. Phys.* **22**, 7169–7192 (2020).

45. C. Bannwarth, S. Ehlert, S. Grimme, GFN2-xTB – An accurate and broadly parametrized self-consistent tight-binding quantum chemical method with multipole electrostatics and density-dependent dispersion contributions. *J. Chem. Theory Comput.* **15**, 1652–1671 (2019).

46. N. M. O'Boyle, M. Banck, C. A. James, C. Morley, T. Vandermeersch, G. R. Hutchison, Open Babel: An open chemical toolbox. *J. Chem.* **3**, 33 (2011).
